# A Meta-analysis of Gene Expression Signatures of Blood Pressure and Hypertension

**DOI:** 10.1371/journal.pgen.1005035

**Published:** 2015-03-18

**Authors:** Tianxiao Huan, Tõnu Esko, Marjolein J. Peters, Luke C. Pilling, Katharina Schramm, Claudia Schurmann, Brian H. Chen, Chunyu Liu, Roby Joehanes, Andrew D. Johnson, Chen Yao, Sai-xia Ying, Paul Courchesne, Lili Milani, Nalini Raghavachari, Richard Wang, Poching Liu, Eva Reinmaa, Abbas Dehghan, Albert Hofman, André G. Uitterlinden, Dena G. Hernandez, Stefania Bandinelli, Andrew Singleton, David Melzer, Andres Metspalu, Maren Carstensen, Harald Grallert, Christian Herder, Thomas Meitinger, Annette Peters, Michael Roden, Melanie Waldenberger, Marcus Dörr, Stephan B. Felix, Tanja Zeller, International Consortium for Blood Pressure GWAS (ICBP), Ramachandran Vasan, Christopher J. O'Donnell, Peter J. Munson, Xia Yang, Holger Prokisch, Uwe Völker, Joyce B. J. van Meurs, Luigi Ferrucci, Daniel Levy

**Affiliations:** 1 The National Heart, Lung, and Blood Institute's Framingham Heart Study, Framingham, Massachusetts, United States of America; 2 The Population Sciences Branch, Division of Intramural Research, National Heart, Lung, and Blood Institute, Bethesda, Maryland, United States of America; 3 Estonian Genome Center, University of Tartu, Tartu, Estonia; 4 Division of Endocrinology, Children’s Hospital Boston, Boston, Massachusetts, United States of America; 5 Department of Genetics, Harvard Medical School, Boston, Massachusetts, United States of America; 6 Broad Institute of Harvard and MIT, Cambridge, Massachusetts, United States of America; 7 Department of Internal Medicine, Erasmus Medical Centre Rotterdam, Rotterdam, The Netherlands; 8 Netherlands Genomics Initiative–sponsored Netherlands Consortium for Healthy Aging (NGI‐NCHA), Leiden and Rotterdam, The Netherlands; 9 Epidemiology and Public Health Group, Medical School, University of Exeter, Exeter, United Kingdom; 10 Institute of Human Genetics, Helmholtz Zentrum München–German Research Center for Environmental Health, Neuherberg, Germany; 11 Institute of Human Genetics, Technische Universität München, München, Germany; 12 Department of Functional Genomics, Interfaculty Institute for Genetics and Functional Genomics, University Medicine Greifswald, Greifswald, Germany; 13 The Charles Bronfman Institute for Personalized Medicine, Genetics of Obesity & Related Metabolic Traits Program, Icahn School of Medicine at Mount Sinai, New York, New York, United States of America; 14 Mathematical and Statistical Computing Laboratory, Center for Information Technology, National Institutes of Health, Bethesda, Maryland, United States of America; 15 Harvard Medical School, Boston, Massachusetts, United States of America; 16 Hebrew SeniorLife, Boston, Boston, Massachusetts, United States of America; 17 Cardiovascular Epidemiology and Human Genomics Branch, Division of Intramural Research, National Heart, Lung and Blood Institute, Bethesda, Maryland, United States of America; 18 Division of Geriatrics and Clinical Gerontology National Institute on Aging, Bethesda, Maryland, United States of America; 19 Genomics Core facility Genetics & Developmental Biology Center, National Heart, Lung, and Blood Institute, Bethesda, Maryland, United States of America; 20 Department of Epidemiology, Erasmus Medical Centre Rotterdam, Rotterdam, The Netherlands; 21 Laboratory of Neurogenetics, National Institute on Aging, Bethesda, Maryland, United States of America; 22 Geriatric Unit, Azienda Sanitaria Firenze, Florence, Italy; 23 Institute for Clinical Diabetology, German Diabetes Center, Leibniz Center for Diabetes Research at Heinrich Heine University Düsseldorf, Düsseldorf, Germany; 24 German Center for Diabetes Research (DZD e.V.), Partner Düsseldorf, Düsseldorf, Germany; 25 Research Unit of Molecular Epidemiology, Helmholtz Zentrum München–German Research Center for Environmental Health, Neuherberg, Germany; 26 Institute of Epidemiology II, Helmholtz Zentrum München—German Research Center for Environmental Health, Neuherberg, Germany; 27 German Center for Diabetes Research (DZD e.V.), Partner Munich, Munich, Germany; 28 DZHK (German Centre for Cardiovascular Research), partner site Munich Heart Alliance, Munich, Germany; 29 Division of Endocrinology and Diabetology, Medical Faculty, Heinrich-Heine University Düsseldorf, Düsseldorf, Germany; 30 University Medicine Greifswald, Department of Internal Medicine B—Cardiology, Greifswald, Germany; 31 DZHK (German Center for Cardiovascular Research), partner site Greifswald, Greifswald, Germany; 32 Universitäres Herzzentrum Hamburg, Hamburg, Germany; 33 DZHK (German Centre for Cardiovascular Research), partner site Hamburg/Kiel/Lübeck, Hamburg, Germany; 34 Department of Integrative Biology and Physiology, University of California, Los Angeles, Los Angeles, California, United States of America; 35 Intramural Research Program, National Institute on Aging, National Institutes of Health, Baltimore, Maryland, United States of America; University of Oxford, UNITED KINGDOM

## Abstract

Genome-wide association studies (GWAS) have uncovered numerous genetic variants (SNPs) that are associated with blood pressure (BP). Genetic variants may lead to BP changes by acting on intermediate molecular phenotypes such as coded protein sequence or gene expression, which in turn affect BP variability. Therefore, characterizing genes whose expression is associated with BP may reveal cellular processes involved in BP regulation and uncover how transcripts mediate genetic and environmental effects on BP variability. A meta-analysis of results from six studies of global gene expression profiles of BP and hypertension in whole blood was performed in 7017 individuals who were not receiving antihypertensive drug treatment. We identified 34 genes that were differentially expressed in relation to BP (Bonferroni-corrected *p*<0.05). Among these genes, *FOS* and *PTGS2* have been previously reported to be involved in BP-related processes; the others are novel. The top BP signature genes in aggregate explain 5%–9% of inter-individual variance in BP. Of note, rs3184504 in *SH2B3*, which was also reported in GWAS to be associated with BP, was found to be a trans regulator of the expression of 6 of the transcripts we found to be associated with BP (*FOS*, *MYADM*, *PP1R15A*, *TAGAP*, *S100A10*, and *FGBP2*). Gene set enrichment analysis suggested that the BP-related global gene expression changes include genes involved in inflammatory response and apoptosis pathways. Our study provides new insights into molecular mechanisms underlying BP regulation, and suggests novel transcriptomic markers for the treatment and prevention of hypertension.

## Author Summary

The focus of blood pressure (BP) GWAS has been the identification of common DNA sequence variants associated with the phenotype; this approach provides only one dimension of molecular information about BP. While it is a critical dimension, analyzing DNA variation alone is not sufficient for achieving an understanding of the multidimensional complexity of BP physiology. The top loci identified by GWAS explain only about 1 percent of inter-individual BP variability. In this study, we performed a meta-analysis of gene expression profiles in relation to BP and hypertension in 7017 individuals from six studies. We identified 34 differentially expressed genes for BP, and discovered that the top BP signature genes explain 5%–9% of BP variability. We further linked BP gene expression signature genes with BP GWAS results by integrating expression associated SNPs (eSNPs) and discovered that one of the top BP loci from GWAS, rs3184504 in *SH2B3*, is a *trans* regulator of expression of 6 of the top 34 BP signature genes. Our study, in conjunction with prior GWAS, provides a deeper understanding of the molecular and genetic basis of BP regulation, and identifies several potential targets and pathways for the treatment and prevention of hypertension and its sequelae.

## Introduction

Systolic and diastolic blood pressure (SBP and DBP) are complex physiological traits that are affected by the interplay of multiple genetic and environmental factors. Hypertension (HTN) is a critical risk factor for stroke, renal failure, heart failure, and coronary heart disease [1]. Genome-wide association studies (GWAS) have identified numerous loci associated with BP traits [2,3]. These loci, however, only explain a small proportion of inter-individual BP variability. In aggregate the 29 loci reported by the International Consortium of Blood Pressure (ICBP) consortium GWAS account for about one percent of BP variation in the general population [3]. Most genes near BP GWAS loci are not known to be mechanistically associated with BP regulation [3]. Therefore, further studies are needed to determine whether the genes implicated in GWAS demonstrate functional relations to BP physiology and to uncover the molecular actions and interactions of genetic and environmental factors involved in BP regulation.

Alterations in gene expression may mediate the effects of genetic variants on phenotype variability. We hypothesized that characterizing gene expression signatures of BP would reveal cellular processes involved in BP regulation and uncover how transcripts mediate genetic and environmental effects on BP variability. We additionally hypothesized that by integrating gene expression profiling with genetic variants associated with altered gene expression (eSNPs or eQTLs) and with BP GWAS results, we would be able to characterize the genetic architecture of gene expression effects on BP regulation.

Several previous studies have examined the association of global gene expression with BP [4,5] or HTN [6,7]. Most of these studies, however, were based on small sample sizes and lacked replication [4,5,6,7]. To address this challenge, we conducted an association study of global gene expression levels in whole blood with BP traits (SBP, DBP, and HTN) in six independent studies. In order to avoid the possibility that the differentially expressed genes we identified reflect drug treatment effects, we excluded individuals receiving anti-hypertensive treatment. The eligible study sample included 7017 individuals: 3679 from the Framingham Heart Study (FHS), 972 from the Estonian Biobank (EGCUT), 604 from the Rotterdam Study (RS) [8], 597 from the InCHIANTI Study, 565 from the Cooperative Health Research in the Region of Augsburg [KORA F4] Study [9], and 600 from the Study of Health in Pomerania [SHIP-TREND] [10]. We first identified differentially expressed BP genes in the FHS (n = 3679) followed by external replication in the other five studies (n = 3338). Subsequently, we performed a meta-analysis of all 7017 individuals from the six studies, and identified 34 differentially expressed genes associated with BP traits using a stringent statistical threshold based on Bonferroni correction for multiple testing of 7717 unique genes. The differentially expressed genes for BP (BP signature genes) were further integrated with eQTLs and with BP GWAS results in an effort to differentiate downstream transcriptomic changes due to BP from putatively causal pathways involved in BP regulation.

## Results

### Clinical characteristics

After excluding individuals receiving anti-hypertensive treatment, the eligible sample size was 7017 (FHS, n = 3679; EGCUT, n = 972; RS, n = 604; InCHIANTI, n = 597; KORA F4, n = 565 and SHIP-TREND, n = 600). Clinical characteristics of participants from the four studies are presented in **Table 1**. The mean age varied across the cohorts (FHS = 51, EGCUT = 36, RS = 58, InCHIANTI = 71, KORA F4 = 72 and SHIP-TREND = 46 years) as did the proportion of individuals with hypertension (11% in FHS, 19% in EGCUT, 35% in RS, 45% in InCHIANTI, 26% in KORA, and 12% in SHIP).

**Table 1:** Clinical characteristics of the study cohorts.

	FHS N = 3,679	EGCUT N = 972	RS N = 604	InCHIANTI N = 597	KORA F4 N = 565	SHIP-TREND N = 600
**Age (yr)**	51 ± 12	36 ± 14	58 ± 8	71 ± 16	72 ± 5	46 ± 13
**Sex, male (%)**	42	49	46	46	51	43
**Hypertension (%)**	11	19	35	45	26	12
**BMI (kg/m** ^**2**^)	27.2 ± 5.3	24.8 ± 4.4	26.8 ± 4.1	27.0 ± 4.2	29.8± 4.6	26 ± 4.2
**Systolic BP (mm Hg)**	118 ± 15	122 ± 16	132 ± 20	132 ± 20	129± 21	120 ± 15
**Diastolic BP (mm Hg)**	74 ±9	76 ± 10	82 ± 11	78 ±10	73±11	75 ± 9

### Identification and replication of differentially expressed BP signature genes

**Fig 1:**
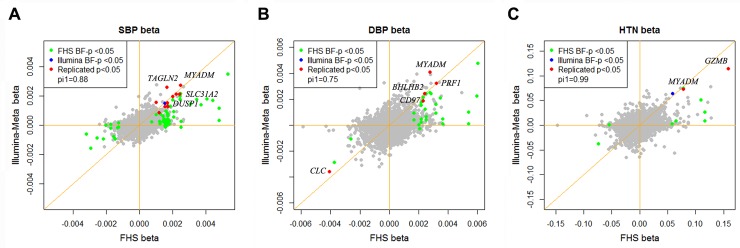
Effect size of differentially expressed BP genes in the Framingham Heart Study and the Illumina cohorts. A) SBP; B) DBP; C) HTN. The x-axis is the effect size of the differentially expressed genes in the FHS cohort and the y-axis is the effect size in the Illumina cohorts. The BP signature genes identified both in the FHS and the Illumina cohorts at *p*<0.05 (Bonferroni corrected) are highlighted. *pi1* values indicate the proportion of significant signals among the tested associations [11] (See details in the Methods section).

At a Bonferroni corrected *p*<0.05, we identified 73, 31, and 8 genes that were differentially expressed in relation to SBP, DBP, and HTN, respectively in the FHS, which used an Affymetrix array for expression profiling, and 6, 1, and 1 genes in the meta-analysis of the 5 cohorts that used an Illumina array (Illumina cohorts): EGCUT, RS, InCHIANTI, KORA F4 and SHIP-TREND (**S1 Table**). For each differentially expressed BP gene in the FHS or in the Illumina cohorts, we attempted replication in the other group. At a replication *p*<0.05 (Bonferroni corrected), 13 unique genes that were identified in the FHS were replicated in the Illumina cohorts, including 10 for SBP (*CD97*, *TAGAP*, *DUSP1*, *FOS*, *MCL1*, *MYADM*, *PPP1R15A*, *SLC31A2*, *TAGLN2*, and *TIPARP*), 5 for DBP (*CD97*, *BHLHE40*, *PRF1*, *CLC*, and *MYADM*), and 2 for HTN (*GZMB* and *MYADM*) (**Table 2**). Each of the unique BP signature genes in the Illumina cohorts, 6 for SBP (*TAGLN2*, *BHLHE40*, *MYADM*, *SLC31A2*, *DUSP1*, and *MCL1*), 1 for DBP (*BHLHE40*) and 1 for HTN (*SLC31A2*), replicated in the FHS. All 6 Illumina cohorts BP signature genes that replicated in the FHS were among the 13 FHS BP signature genes that replicated in the Illumina cohorts. The BP signature genes identified in the FHS showed enrichment in the Illumina cohorts at *pi1* = 0.88, 0.75, and 0.99 for SBP, DBP, and HTN respectively (*pi1* value indicates the proportion of significant signals among the tested associations [11]; see details in the Methods section). **Fig. 1** shows that the mean gene expression levels of the top BP signature genes were consistent with the BP phenotypic changes observed in the FHS and the Illumina cohorts.

**Table 2:** Differentially expressed genes associated with BP and hypertension at Bonferroni correction *p*<0.05 in meta-analysis of the six cohorts.

Gene	Chr.	Gene Description	FHS Beta	FHS s.e.	FHS pvalue	Illumina Beta	Illumina s.e.	Illumina pvalue	Meta *	Meta s.e.	Meta pvalue
—***SBP Signature genes***											
SLC31A2	9	solute carrier family 31 (copper transporters), member 2	2.4E-03	3.3E-04	1.2E-13	2.1E-03	3.3E-04	9.9E-11	2.3E-03	2.3E-04	<1E-16
MYADM	19	myeloid-associated differentiation marker	2.5E-03	3.2E-04	2.2E-14	2.7E-03	3.9E-04	2.2E-12	2.6E-03	2.5E-04	<1E-16
DUSP1	5	dual specificity phosphatase 1	2.2E-03	3.9E-04	1.1E-08	2.1E-03	4.2E-04	3.7E-07	2.2E-03	2.9E-04	2.0E-14
TAGLN2	1	transgelin 2	2.0E-03	4.1E-04	1.0E-06	2.0E-03	4.0E-04	1.3E-06	2.0E-03	2.9E-04	5.8E-12
CD97	19	CD97 molecule	1.7E-03	3.2E-04	1.4E-07	1.5E-03	3.5E-04	1.6E-05	1.6E-03	2.4E-04	1.0E-11
BHLHE40	3	basic helix-loop-helix family, member e40	1.5E-03	3.4E-04	4.3E-06	1.5E-03	3.0E-04	6.4E-07	1.5E-03	2.2E-04	1.2E-11
MCL1	1	myeloid cell leukemia sequence 1 (BCL2-related)	1.0E-03	2.0E-04	7.5E-07	1.6E-03	3.2E-04	1.5E-06	1.2E-03	1.7E-04	1.4E-11
PRF1	10	perforin 1 (pore forming protein)	2.5E-03	4.1E-04	2.5E-09	1.8E-03	5.3E-04	1.0E-03	2.2E-03	3.3E-04	1.6E-11
GPR56	16	G protein-coupled receptor 56	2.0E-03	3.4E-04	3.5E-09	1.7E-03	5.8E-04	3.0E-03	1.9E-03	2.9E-04	3.9E-11
PPP1R15A	19	protein phosphatase 1, regulatory (inhibitor) subunit 15A	1.5E-03	2.6E-04	1.7E-09	1.3E-03	3.0E-04	2.8E-05	1.4E-03	2.4E-04	1.5E-08
FGFBP2	4	fibroblast growth factor binding protein 2	2.3E-03	5.0E-04	5.8E-06	2.0E-03	6.2E-04	1.5E-03	2.2E-03	3.9E-04	3.3E-08
GNLY	2	granulysin	2.6E-03	6.4E-04	3.6E-05	2.6E-03	7.2E-04	3.0E-04	2.6E-03	4.8E-04	4.0E-08
FOS	14	FBJ murine osteosarcoma viral oncogene homolog	1.7E-03	2.5E-04	1.6E-11	2.6E-03	6.3E-04	3.6E-05	2.3E-03	4.1E-04	4.8E-08
NKG7	19	natural killer cell group 7 sequence	2.3E-03	5.3E-04	1.9E-05	1.4E-03	5.5E-04	8.8E-03	1.9E-03	3.8E-04	9.4E-07
GRAMD1A	19	GRAM domain containing 1A	-6.0E-04	1.4E-04	2.1E-05	-6.7E-04	2.8E-04	1.8E-02	-6.2E-04	1.3E-04	1.1E-06
GLRX5	14	glutaredoxin 5	1.7E-03	3.9E-04	1.3E-05	1.3E-03	6.1E-04	3.5E-02	1.6E-03	3.3E-04	1.5E-06
TMEM43	3	transmembrane protein 43	7.5E-04	2.1E-04	3.0E-04	7.7E-04	2.5E-04	2.4E-03	7.6E-04	1.6E-04	2.3E-06
TIPARP	3	TCDD-inducible poly(ADP-ribose) polymerase	1.2E-03	2.3E-04	1.3E-07	8.6E-04	2.4E-04	3.3E-04	9.5E-04	2.0E-04	2.6E-06
AHNAK	11	AHNAK Nucleoprotein	9.1E-04	2.6E-04	4.1E-04	9.7E-04	3.4E-04	4.0E-03	9.3E-04	2.0E-04	5.2E-06
PIGB	15	phosphatidylinositol glycan anchor biosynthesis, class B	1.1E-03	3.1E-04	5.3E-04	6.7E-04	2.1E-04	1.9E-03	8.0E-04	1.8E-04	6.1E-06
TAGAP	6	T-cell activation RhoGTPase activating protein	1.7E-03	2.5E-04	5.7E-12	1.3E-03	3.7E-04	7.1E-04	1.4E-03	3.1E-04	6.4E-06
—***DBP Signature genes***											
BHLHE40	3	basic helix-loop-helix family, member e40	2.4E-03	5.1E-04	2.3E-06	2.5E-03	5.2E-04	2.8E-06	2.4E-03	3.6E-04	2.7E-11
ANXA1	9	annexin A1	3.5E-03	5.7E-04	1.2E-09	2.1E-03	7.8E-04	6.3E-03	3.0E-03	4.6E-04	6.5E-11
PRF1	10	perforin 1 (pore forming protein)	3.2E-03	6.2E-04	3.2E-07	3.2E-03	9.4E-04	5.7E-04	3.2E-03	5.2E-04	6.7E-10
KCNJ2	17	potassium inwardly-rectifying channel, subfamily J, member 2	-2.6E-03	5.6E-04	3.9E-06	-2.0E-03	5.5E-04	2.6E-04	-2.3E-03	3.9E-04	4.9E-09
CLC	19	Charcot-Leyden crystal protein	-4.1E-03	8.6E-04	2.6E-06	-3.6E-03	1.0E-03	5.7E-04	-3.9E-03	6.7E-04	5.8E-09
CD97	19	CD97 molecule	2.3E-03	4.8E-04	1.6E-06	1.9E-03	5.8E-04	1.1E-03	2.1E-03	3.7E-04	7.4E-09
IL2RB	22	interleukin 2 receptor, beta	2.3E-03	4.9E-04	3.0E-06	2.2E-03	7.3E-04	2.4E-03	2.3E-03	4.1E-04	2.5E-08
S100A10	1	S100 calcium binding protein A10	3.2E-03	6.1E-04	2.4E-07	1.6E-03	6.2E-04	9.9E-03	2.4E-03	4.4E-04	4.0E-08
GPR56	16	G protein-coupled receptor 56	2.5E-03	5.2E-04	1.1E-06	2.4E-03	1.0E-03	1.7E-02	2.5E-03	4.6E-04	5.5E-08
TIPARP	3	TCDD-inducible poly(ADP-ribose) polymerase	1.3E-03	3.4E-04	1.3E-04	1.1E-03	3.1E-04	2.8E-04	1.2E-03	2.3E-04	1.4E-07
HAVCR2	5	Hepatitis A Virus Cellular Receptor 2	1.7E-03	4.6E-04	3.8E-04	1.8E-03	4.8E-04	1.8E-04	1.7E-03	3.3E-04	2.4E-07
PTGS2	1	prostaglandin-endoperoxide synthase 2 (prostaglandin G/H synthase and cyclooxygenase)	-2.1E-03	4.9E-04	2.2E-05	-1.3E-03	5.1E-04	9.0E-03	-1.7E-03	3.5E-04	1.0E-06
MYADM	19	myeloid-associated differentiation marker	2.8E-03	4.9E-04	1.7E-08	4.1E-03	1.0E-03	8.6E-05	3.6E-03	7.4E-04	1.1E-06
ANTXR2	4	anthrax toxin receptor 2	1.5E-03	3.3E-04	5.2E-06	8.3E-04	4.3E-04	5.5E-02	1.3E-03	2.6E-04	1.7E-06
OBFC2A	2	nucleic acid binding protein 1	-1.7E-03	3.9E-04	7.2E-06	-9.6E-04	4.6E-04	3.8E-02	-1.4E-03	3.0E-04	1.8E-06
GRAMD1A	19	GRAM domain containing 1A	-9.3E-04	2.1E-04	1.4E-05	-8.7E-04	5.0E-04	7.8E-02	-9.2E-04	2.0E-04	2.8E-06
ARHGAP15	2	Rho GTPase activating protein 15	-1.3E-03	4.1E-04	1.1E-03	-1.4E-03	4.4E-04	1.5E-03	-1.4E-03	3.0E-04	5.2E-06
FBXL5	4	F-box and leucine-rich repeat protein 5	-1.6E-03	3.7E-04	2.1E-05	-9.4E-04	4.9E-04	5.5E-02	-1.3E-03	2.9E-04	5.3E-06
SLC31A2	9	solute carrier family 31 (copper transporters), member 2	2.8E-03	4.9E-04	1.0E-08	2.4E-03	8.1E-04	2.6E-03	2.6E-03	5.6E-04	5.4E-06
VIM	10	vimentin	1.7E-03	3.8E-04	5.5E-06	7.6E-04	5.9E-04	2.0E-01	1.4E-03	3.2E-04	6.2E-06
—***HTN Signature genes***											
SLC31A2	9	solute carrier family 31 (copper transporters), member 2	5.9E-02	1.4E-02	1.9E-05	6.4E-02	1.4E-02	2.1E-06	6.1E-02	9.6E-03	1.8E-10
MYADM	19	myeloid-associated differentiation marker	7.8E-02	1.4E-02	1.2E-08	7.3E-02	2.1E-02	6.2E-04	7.4E-02	1.4E-02	3.0E-07
TAGAP	6	T-cell activation RhoGTPase activating protein	4.4E-02	1.1E-02	3.2E-05	3.2E-02	1.2E-02	5.3E-03	3.9E-02	7.8E-03	7.3E-07
GZMB	14	granzyme B (granzyme 2, cytotoxic T-lymphocyte-associated serine esterase 1)	1.6E-01	2.3E-02	1.1E-11	1.1E-01	3.5E-02	9.6E-04	1.3E-01	2.6E-02	1.4E-06
KCNJ2	17	potassium inwardly-rectifying channel, subfamily J, member 2	-5.2E-02	1.6E-02	8.4E-04	-4.4E-02	1.3E-02	5.5E-04	-4.7E-02	9.9E-03	1.7E-06

*Meta: meta-analysis of all six cohorts.

The 73 SBP signature genes in the FHS (55 of these 73 genes were measured in the Illumina cohorts) at a Bonferroni corrected *p*<0.05 in aggregate explained 9.4% of SBP phenotypic variance in the Illumina cohorts, and the 31 DBP signature genes from the FHS (22 of these 31 genes were measured in the Illumina cohorts) in aggregate explained 5.3% of DBP phenotypic variance in the Illumina cohorts. These results suggest that in contrast to common genetic variants identified by BP GWAS, which explain in aggregate only about 1% of inter-individual BP variation [3], changes in gene expression levels explains a considerably larger proportion of phenotypic variance in BP.

### Meta-analysis of the six cohorts identifies differentially expressed BP signature genes

A meta-analysis of differential expression across all six cohorts revealed 34 differentially expressed BP genes at *p*<0.05 (Bonferroni corrected for 7717 genes that were measured and passed quality control in the FHS and Illumina cohorts), including 21 for SBP, 20 for DBP, and 5 for HTN (**Table 2** and **S2 Fig.**). All of the 34 differentially expressed BP signature genes showed directional consistency in the FHS and the Illumina cohorts (**Table 2**). The 34 BP signature genes included all 13 genes that were cross-validated between the FHS and the Illumina cohorts. Of the 34 BP signature genes, 27 were positively correlated with BP and only 7 genes were negatively correlated. *MYADM* and *SLC31A2* were top signature genes for SBP, DBP, and HTN. At FDR<0.2, 224 unique genes were differentially expressed in relation BP phenotypes including 142 genes for SBP, 137 for DBP, and 45 for HTN (details are reported in the **S1–S2 Text**, and **S3–S5 Table**).

### Functional analysis of differentially expressed BP signature genes

We used gene set enrichment analysis (GSEA) to identify the biological process and pathways associated with gene expression changes in relation to SBP, DBP, and HTN in order to better understand the biological themes within the data. As shown in **Table 3**, the GSEA of genes whose expression was positively associated with BP showed enrichment for antigen processing and presentation (*p*<0.0001), apoptotic program (*p*<0.0001), inflammatory response (*p*<0.0001), and oxidative phosphorylation (*p* = 0.0018). The negatively associated genes showed enrichment for nucleotide metabolic process (*p*<0.0001), positive regulation of cellular metabolic process (*p*<0.0001), and positive regulation of DNA dependent transcription (*p* = 0.0021).

**Table 3:** Gene set enrichment analysis for BP associated gene expression changes.

Name	Pos / Neg associated gene expression changes	Database	Number of genes in pathway	NES*	p value	FDR
- ***DBP signature***						
Antigen processing and presentation	Positive	KEGG	37	2.0	<1E-4	0.01
Nature killer cell mediated cytotoxicity	Positive	KEGG	71	1.8	<1E-4	0.07
Porphyrin and chlorophyll metabolism	Positive	KEGG	15	1.7	0.01	0.13
Rho protein signaling transduction	Negative	GO-BP	18	-1.8	3.9E-3	0.10
Receptor mediated endocytosis	Negative	GO-BP	16	-1.8	3.9E-3	0.17
Detection of stimulus	Negative	GO-BP	18	-1.9	9.8E-3	0.20
- ***SBP signature***						
Natural killer cell mediated cytotoxicity	Positive	KEGG	71	1.9	1.7E-3	0.05
Apoptotic program	Positive	GO-BP	37	1.9	<1E-4	0.03
Inflammatory response	Positive	GO-BP	72	2.0	<1E-4	0.05
Nucleotide metabolic process	Negative	GO-BP	32	-1.9	<1E-4	0.04
Translation	Negative	GO-BP	79	-1.8	<1E-4	0.05
- ***HTN signature***						
Antigen processing and presentation	Positive	KEGG	37	1.8	<1E-4	0.04
Oxidative phosphorylation	Positive	KEGG	52	1.8	1.8E-3	0.05
Apoptotic program	Positive	GO-BP	37	1.9	1.8E-3	0.14
Positive regulation of nucleic acid metabolic process	Negative	GO-BP	71	-1.9	<1E-4	0.08
Positive regulation of cellular metabolic process	Negative	GO-BP	105	-1.8	<1E-4	0.08
Positive regulation of transcription DNA dependent	Negative	GO-BP	56	-1.8	2.1E-3	0.09

*NES: normalized enrichment score;

**GO-BP**: Gene ontology- biological process;

**KEGG**: Kyoto encyclopedia of genes and genomes.

### Genetic effects on expression of BP signature genes

**Fig 2:**
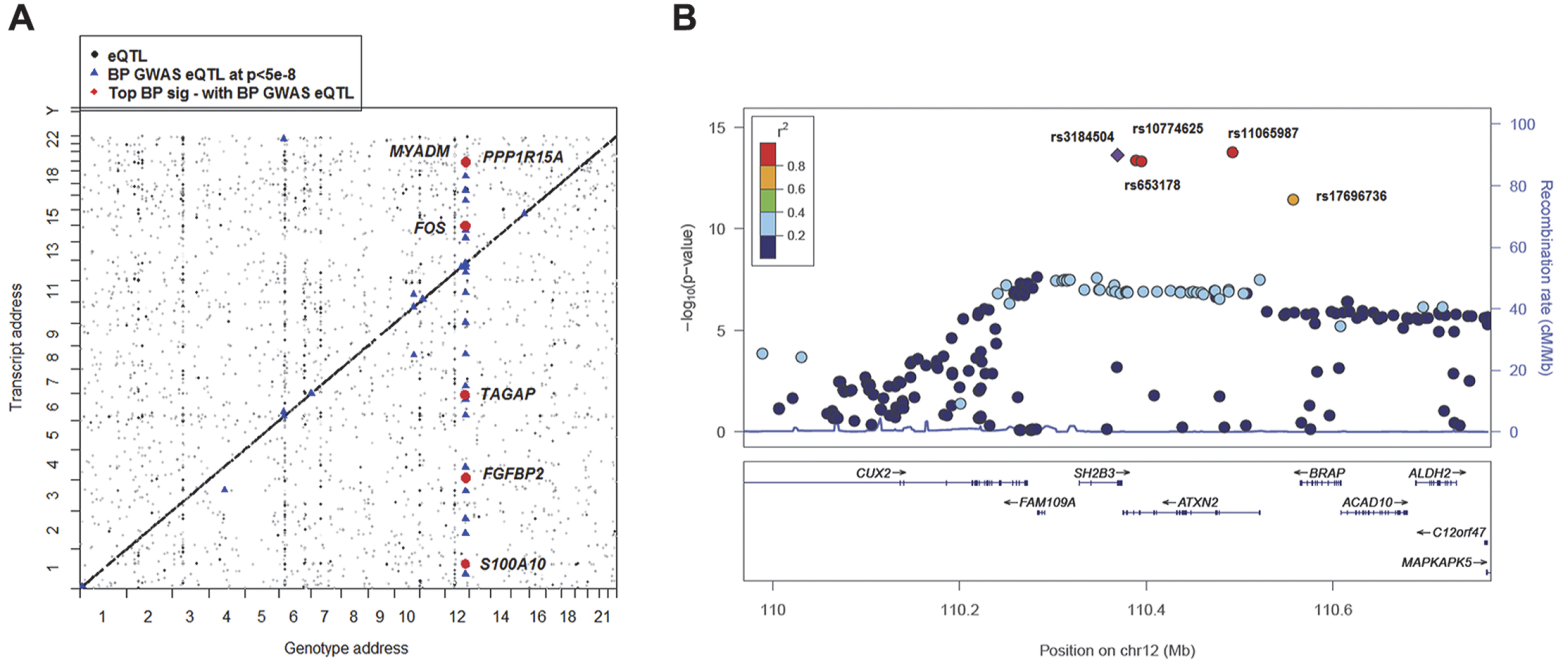
Global view of BP eQTLs effects on differentially expressed BP signature genes. A) 2-Dimensional plot of in whole blood eQTLs vs. transcript position genome wide. eQTL-transcript pairs at FDR<0.1 are shown in black dots; those that fall along the diagonal are cis eQTLs and all others are trans eQTLS. eQTL-transcript pair SNPs that are associated with BP in GWAS [3] are highlighted with blue triangles. eQTL-transcript pair genes that are BP signature genes from analysis of differential gene expression in relation to BP are depicted by red circles. B) Regional association plots for rs3184504 proxy QTLs that showing association with BP in ICBP GWAS [3]. −log10(p) indicated the −log10 transformed DBP association *p* values in ICBP GWAS [3]. Color coding indicates the strength (measured by r^2^) of LD of each SNP with the top SNP (rs3184504). Five master *trans-*eQTLs (also BP GWAS SNPs) for BP signature genes are labeled in the figure. This figure was drawn by LocusZoom [32].

Among the 34 BP signatures genes from the meta-analysis of all 6 studies, 33 were found to have *cis-*eQTLs and 26 had *trans-*eQTLs (**Fig. 2A** and **S2 Table**) based on whole blood profiling [12,13]. Of these, six master *trans-*eQTLs mapped to either five or six BP signature genes (no master *cis-*eQTL was identified). Five master *trans-*eQTLs (rs653178, rs3184504, rs10774625, rs11065987, and rs17696736) were located on chromosome *12q24* within the same linkage disequilibrium (LD) block (r^2^ >0.8, **Fig. 2B**). We retrieved a peak *cis-* and *trans-*eQTL for each BP signature gene. The peak *cis-*eQTL explained 0.2–20% of the variance in the corresponding transcript levels, in contrast, the peak *trans-*eQTL accounted for very little (0.02–2%) of the corresponding transcript variance. Westra *et al*. also reported a similar small proportion of variance in transcript levels explained by *trans-*eQTLs [12].

We then linked the *cis-* and *trans-*eQTLs of the 34 BP signature genes with BP GWAS results from the ICBP Consortium [3] and the NHGRI GWAS Catalog [14] (**Fig. 2** and **S2 Table**). We did not find any *cis-*eQTLs for the top BP signature genes that also were associated with BP in the ICBP GWAS [3]. However, the 6 master *trans-*eQTLs were all associated with BP at *p*<5e-8 in the ICBP GWAS [3] and were associated with multiple complex diseases or traits (**Table 4**). For example, rs3184504, a nonsynonymous SNP in *SH2B3* that was associated in GWAS with BP, coronary heart disease, hypothyroidism, rheumatoid arthritis, and type 1 diabetes [12], is a *trans-*eQTL for 6 of our 34 BP signature genes from the meta-analysis (*FOS*, *MYADM*, *PP1R15A*, *TAGAP*, *S100A10*, and *FGBP2*; **Fig. 2A-B** and **Table 4**). These 6 genes are all highly expressed in neutrophils, and their expression levels are correlated significantly (average r^2^ = 0.04, *p*<1e-16). rs653178, intronic to *ATXN2* and in perfect LD with rs3184504 (r^2^ = 1), also is associated with BP and multiple other diseases in the NHGRI GWAS Catalog [14]. It also is a *trans-*eQTL for the same 6 BP signature genes (**Table 4**). These two SNPs are *cis-*eQTLs for expression *SH2B3* in whole blood (FDR<0.05), but not for *ATXN2* (FDR = 0.4). We found that the expression of *SH2B3* is associated with expression of *MYADM*, *PP1R15A*, and *TAGAP* (at Bonferroni corrected *p*<0.05), but not with *FOS*, *S100A10*, or *FGBP2*. The expression of *ATXN2* was associated with expression of 5 of the 6 genes (*PP1R15A* was not associated). **S3 Fig.** shows the coexpression levels of the eight genes that were *cis-* or *trans-* associated with rs3184504 and rs653178 genotypes. These results suggest that there may be a pathway or gene co-regulatory mechanism underling BP regulation involving these genes that is driven by this common genetic variant (rs3184504; minor allele frequency 0.47) or its proxy SNPs.

**Table 4:** GWAS eQTLs for the top differentially expressed BP signature genes.

		SNP—Trait Association			SNP-Gene Association			Gene-Trait Association		
SNP ID	SNP. Location	ICBP-SBP pval	ICBP-DBP pval	Other Traits in GWAS Catalog	Gene	Chr. Gene	Cis/Trans	SBP pval	DBP pval	HTN pval
rs3184504*	chr12 (missense, SH2B3)	1.70E-09	2.30E-14	Coronary heart disease; Rheumatoid arthritis; Type 1 diabetes	MYADM	chr19	trans	**<1e-16** ^&^	**1.1e-6**	**3.0e-7**
					FOS	chr14	trans^§^	**4.9e-8**	3.2e-4	7.9e-5
					PPP1R15A	chr19	trans^§^	**1.6e-8**	1.2e-5	6.1e-4
					TAGAP	chr6	trans	**6.4e-6**	1.3e-4	**7.3e-7**
					S100A10	chr1	trans^§^	2.6e-4	**4.0e-8**	7.0e-5
					FGFBP2	chr4	trans^§^	**3.3e-8**	1.8e-5	5.1e-3
rs10187424	chr2 (intergenic)	-	-	Prostate cancer	GNLY	chr2	cis^§^	**4.0e-8**	2.8e-5	2.2e-4
rs411174	chr5 (intron, ITK)	-	-	Personality dimensions	HAVCR2	chr5	cis^§^	1.6e-4	**2.4e-7**	1.5e-3
rs3758354	chr9 (intergenic)	-	-	Schizophrenia, bipolar disorder and depression	ANXA1	chr9	cis	1.8e-3	**6.5e-11**	7.5e-3
rs1950500	chr14 (intergenic)	-	-	Height	GZMB	chr14	cis	7.8e-5	6.0e-5	**1.4e-6**
rs8017377	chr14 (missense, NYNRIN)	-	-	LDL cholesterol	GZMB	chr14	cis	7.8e-5	6.0e-5	**1.4e-6**
rs8192917	chr14 (missense, GZMB)	-	-	Vitiligo	GZMB	chr14	cis	7.8e-5	6.0e-5	**1.4e-6**
rs2284033	chr22 (intron, IL2RB)	-	-	Asthma	IL2RB	chr22	cis^§^	1.6e-4	**2.5e-8**	9.3e-3
rs11724635^+^	chr4 (intergenic)	-	-	Parkinsons disease	FBXL5	chr4	cis	5.9e-5	**5.3e-6**	0.07
rs4333130^$^	chr4 (intron, ANTXR2)	-	-	Ankylosing spondylitis	ANTXR2	chr4	cis	2.8e-4	**1.7e-6**	0.04
rs8005962	chr14 (intergenic)	-	-	Tuberculosis	GLRX5	chr14	cis	**1.5e-6**	0.13	0.09
rs7995215	chr13 (intron, GPC6)	-	-	Attention deficit hyperactivity disorder	TAGAP	chr6	trans	**6.4e-6**	1.3e-4	**7.3e-7**
rs12047808	chr1 (intron, C1orf125)	-	-	Multiple sclerosis (age of onset)	FOS	chr14	trans^§^	**4.9e-8**	3.2e-4	7.9e-5
rs2894207	chr6 (intergenic)	-	-	Nasopharyngeal carcinoma	AHNAK	chr11	trans	**5.2e-6**	6.8e-5	1.8e-3
rs3763313	chr6 (neargene 5, BTNL2)	-	-	HIV-1 control	PPP1R15A	chr19	trans	**1.6e-8**	1.2e-5	6.1e-4
rs9376092	chr6 (intergenic)	-	-	Beta thalassemia/hemoglobin E disease	GPR56	Chr16	trans	**3.9e-11**	**5.5e-8**	4.9e-4

* rs653178, intronic to *ATXN2* and in tight linkage disequilibrium with rs3184504 (r^2^ = 1), was also associated with BP in ICBP GWAS and all the 6 genes;

^+^ A proxy SNP rs4698412 at LD r^2^ = 1 associated with the same trait;

$ A proxy SNP rs4389526 at LD r^2^ = 1 associated with the same trait;

^§^ indicated eQTL were identified from[12].

^&^ highlighted p values indicated passing transcriptome-wide significance at Bonferroni corrected *p*<0

We further checked whether the *cis-* or *trans-*eQTLs for the top 34 BP signature genes are associated with other diseases or traits in the NHGRI GWAS catalog [14]. We identified 12 *cis-*eQTLs (for 8 genes) and 6 *trans-*eQTLs (for 6 genes) that are associated with other diseases or traits in the NHGRI GWAS catalog [14] (**Table 4**).

## Discussion

Our meta-analysis of gene expression data from 7017 individuals from six studies identified and characterized whole blood gene expression signatures associated with BP traits. Thirty-four BP signature genes were identified at Bonferroni corrected *p*<0.05 (224 genes were identified at FDR<0.2, reported in the **S1 Text**). Thirteen BP signature genes replicated between the FHS and Illumina cohorts. The top BP signature genes identified in the FHS (55 genes for SBP and 22 genes for DBP) explained 5–9% of interindividual variation in BP in the Illumina cohorts on average.

Among the 34 BP signature genes (at Bonferroni corrected *p*<0.05), only *FOS* [15] and *PTGS2 [16]* have been previously implicated in hypertension. We did not find literature support for a direct role of the remaining signature genes in BP regulation. However, we found several genes involved in biological functions or processes that are highly related to BP, such as cardiovascular disease (*GZMB*, *ANXA1*, *TMEM43*, *FOS*, *KCNJ2*, *PTGS2*, and *MCL1*), angiogenesis (*VIM* and *TIPARP*), and ion channels (*CD97*, *ANXA1*, *S100A10*, *PRF1*, *ANTXR2*, *SLC31A2*, *TIPARP*, and *KCNJ2*). We speculate that these genes may be important for BP regulation, but further experimental validation is needed.

Seven of the 34 signature genes, including *KCNJ2*, showed negative correlation of expression with BP. *KCNJ2* is a member of the potassium inwardly-rectifying channel subfamily; it encodes the inward rectifier K+ channel Kir2.1, and is found in cardiac, skeletal muscle, and nervous tissue [17]. Most outward potassium channels are positively correlated with BP. Loss-of-function mutations in *ROMK* (*KCNJ1*, the outward potassium channel) are associated with Bartter's syndrome, and *ROMK* inhibitors are used in the treatment of hypertension [18,19]. Previous studies reported that greater potassium intake is associated with lower blood pressure [20,21,22,23]. These data suggest that *KCNJ2* up-regulation may be a means of lowering BP.

By linking the BP signature genes with eQTLs and with BP GWAS results, we found several SNPs that are associated with BP in GWAS and that also are *trans* associated with several of our top BP signature genes. For example, rs3184504, a non-synonymous SNP located in exon 3 of *SH2B3*, is associated in GWAS with BP, coronary heart disease, hypothyroidism, rheumatoid arthritis, and type I diabetes [12]. rs3184504 is a common genetic variant with a minor allele frequency of approximately 0.47; the rs3184504-T allele is associated with an increment of 0.58 mm Hg in SBP and of 0.48 mm Hg in DBP [2]. rs3184504 is a *cis-*eQTL for *SH2B3*, expression of this gene was not associated with BP or hypertension in our data. However, rs3184504 also is a *trans-*eQTL for 6 of our 34 BP signature genes: *FOS*, *MYADM*, *PP1R15A*, *TAGAP*, *S100A10*, and *FGBP2*. These 6 genes are highly expressed in neutrophils [12], and are coexpressed. Prior studies have suggested an important role of neutrophils in BP regulation [24]. We speculate that these 6 BP signature genes, all driven by the same BP-associated eQTL, point to a critical and previously unrecognized mechanism involved in BP regulation. Further experimental validation is needed.

One limitation of our study is the use of whole blood derived RNA for transcriptomic profiling. GSEA showed that the top enriched biological processes for the differentially expressed BP genes include inflammatory response. Numerous studies have shown links between inflammation and hypertension [25,26,27]. The top ranked genes in inflammatory response categories provide a guide for further experimental work to recognize the contributions of inflammation to alterations in BP regulation. We speculate that using similar approaches in other tissues might identify additional differentially expressed BP signature genes.

In conclusion, we conducted a meta-analysis of global gene expression profiles in relation to BP and identified a number of credible gene signatures of BP and hypertension. Our integrative analysis of GWAS and gene expression in relation to BP can help to uncover the genetic and genomic architecture of BP regulation; the BP signature genes we identified may represent an early step toward improvements in the detection of susceptibility, and in the prevention and treatment of hypertension.

## Materials and Methods

### Study population and ethics statement

This investigation included six studies (the Framingham Heart Study (FHS), the Estonian Biobank (EGCUT), the Rotterdam Study (RS) [8], the InCHIANTI Study, the Cooperative Health Research in the Region of Augsburg (KORA F4) Study [9], and the Study of Health in Pomerania (SHIP-TREND) [10], each of which conducted genome-wide genotyping, mRNA expression profiling, and had extensive BP phenotype data. Each of the six studies followed the recommendations of the Declaration of Helsinki. The FHS: Systems Approach to Biomarker Research (SABRe) in cardiovascular disease is approved under the Boston University Medical Center’s protocol H-27984. Ethical approval of EGCUT was granted by the Research Ethics Committee of the University of Tartu (UT REC). Ethical approval of the InCHIANTI study was granted by the Instituto Nazionale Riposo e Cura Anziani institutional review board in Italy. Ethical approval of RS was granted by the medical ethics committee of the Erasmus Medical Center. The study protocol of SHIP-TREND was approved by the medical ethics committee of the University of Greifswald. KORA F4 is a population-based survey in the region of Augsburg in Southern Germany which was performed between 2006 and 2008. KORA F4 was approved by the local ethical committees. Informed consent was obtained from each study participant.

Hypertension (HTN) was defined as SBP ≥140 mm Hg or DBP ≥90 mm Hg. We excluded individuals receiving anti-hypertensive treatment because of the possibility that some of the differentially expressed genes we identified would reflect treatment effects. The eligible study sample included 7017 individuals: 3679 from FHS, 972 from EGCUT, 604 from RS, 597 from InCHIANTI, 565 from KORA F4, and 600 from SHIP-TREND.

### Gene expression profiling

RNA was isolated from whole blood samples that were collected in PaxGene tubes (PreAnalytiX, Hombrechtikon, Switzerland) in FHS, RS, InCHIANTI, KORA F4 and SHIP-TREND, and in Blood RNA Tubes (Life Technologies, NY, USA) in EGCUT. Gene expression in the FHS samples used the Affymetrix Exon Array ST 1.0. EGCUT, RS, InCHANTI, KORA F4, and SHIP-TREND used the Illumina HT12v3 (EGCUT, InCHANTI, KORA F4, and SHIP-TREND) or HT12v4 (RS) array. Raw data from gene expression profiling are available online (FHS [http://www.ncbi.nlm.nih.gov/gap; accession number phs000007], EGCUT [GSE48348], RS [GSE33828], InCHIANTI [GSE48152], KORA F4 [E-MTAB-1708] and SHIP-TREND [GSE36382]). The details of sample collection, microarrays, and data processing and normalization in each cohort are provided in the **S2 Text**.

### Identification and replication of differentially expressed genes associated with BP

The association of gene expression with BP was analyzed separately in each of the six studies (Equation 1). A linear mixed model was used in the FHS in order to account for family structure. Linear regression models were used in the other five studies. In each study, gene expression level, denoted by geneExp, was included as the dependent variable, and explanatory variables included blood pressure phenotypes (SBP, DBP, and HTN), and covariates included age, sex, body mass index (BMI), cell counts, and technical covariates. A separate regression model was fitted for each gene. The general formula is shown below, and the details of analyses for each study are provided in the **S2 Text** and **S6 Table**.

$$\mathrm{geneExp}=BP+\sum_{j=1}^{m}\mathrm{covariates}$$

The overall analysis framework is provided in **S1 Fig.**. We first identified differentially expressed genes associated with BP (BP signature genes) in the FHS samples (Set 1) and attempted replication in the meta-analysis results from the Illumina cohorts (Set 2, see Methods, Meta-analysis). We next identified BP signature genes in the Illumina cohorts (Set 2), and then attempted replication in the FHS samples (Set 1). The significance threshold for pre-selecting BP signature genes in discovery was at Bonferroni corrected *p* = 0.05 (in FHS, corrected for 17,318 measured genes [17,873 transcripts], and in illumina cohorts, corrected for 12,010 measured genes [14,222 transcripts] that passed quality control). Replication was established at Bonferroni corrected *p* = 0.05, correcting for the number of pre-selected BP signatures genes in the discovery set. We computed the *pi1* value to estimate the enrichment of significant *p* values in the replication set (the Illumina cohorts) for BP signatures identified in the discovery set (the FHS) by utilizing the R package *Qvalue* [11]. *Pi1* is defined as *1-pi0*. *Pi0* value provided by the *Qvalue* package, represents overall probability that the null hypothesis is true. Therefore, *pi1* value represents the proportion of significant results. For genes passing Bonferroni corrected *p*<0.05 in the discovery set for SBP, DBP and HTN, we calculated *pi1* values for each gene set in the replication set.

### Meta-analysis

We performed meta-analysis of the five Illumina cohorts (for discovery and replication purposes), and then performed meta-analysis of all six cohorts. An inverse variance weighted meta-analysis was conducted using fixed-effects or random-effects models by the *metagen()* function in the R package Meta (http://cran.r-project.org/web/packages/meta/index.html). At first, we tested heterogeneity for each gene using Cochran’s Q statistic. If the heterogeneity p value is significant (*p*<0.05), we will use a random-effects model for the meta-analysis, otherwise use a fixed-effects model. The Benjamini-Hochberg (BH) method [28] was used to calculate FDR for differentially expressed genes in relation to BP following the meta-analysis of all six cohorts. We also used a more stringent threshold to define BP signature genes by utilizing *p*<6.5e-6 (Bonferroni correction for 7717 unique genes [7810 transcript] based on the overlap of FHS and illumina cohort interrogated gene sets).

### Estimating the proportion of variance in BP attributable to BP signature genes

To estimate the proportion of variances in SBP or DBP explained by a group of differentially expressed BP signature genes (gene 1, gene 2, …, gene n), we used the following two models:

Full model:

$$BP=\sum_{i=1}^{n}\mathrm{gene}\;i+\sum_{j=1}^{m}\mathrm{covariates}$$

Null model:

$$BP=\sum_{j=1}^{m}\mathrm{covariates}$$

The proportion of variance in BP attributable to the group of differentially expressed BP signature genes ($h_{BP\_sig}^{2}$) was calculated as:

$$h_{BP\_sig}^{2}=\text{max}\left(0,\frac{\sigma_{G.null}^{2}+\sigma_{err.null}^{2}-\sigma_{G.full}^{2}-\sigma_{err.full}^{2}}{\sigma_{BP}^{2}}\right)$$

where $\sigma_{BP}^{2}$ is the total phenotypic variance of SBP or DBP, $\sigma_{G.full}^{2}$ and $\sigma_{err.full}^{2}$ are the variance and error variance when modeling with the tested group of gene expression traits (gene 1, gene 2, …, gene n), and $\sigma_{G.null}^{2}$ and $\sigma_{err.null}^{2}$ are the variance and error variance when modeling without the tested group of gene expression traits.

The proportion of the variance in BP phenotypes attributable to the FHS BP signature genes was estimated in the five Illumina cohorts, respectively, and then the average proportion values were reported. In turn, the proportion of the variance in BP phenotypes attributable to the Illumina BP signature genes was estimated in the FHS.

### Identifying eQTLs and estimating the proportion of variance in gene expression attributable to single *cis-* or *trans-*eQTLs

SNPs associated with altered gene expression (i.e. eQTLs) were identified using genome-wide genotype and gene expression data in all available FHS samples (n = 5257) at FDR<0.1 (Joehanes R, submitted, 2014, and a brief summary of methods and results are provided in the **S2 Text**). A *cis-*eQTL was defined as an eQTL within 1 megabase (MB) flanking the gene. Other eQTLs were defined as *trans-*eQTLs. We combined the eQTL list generated in the FHS with the eQTLs generated by meta-analysis of seven other studies (n = 5300) that were also based on whole blood expression[12].

For every BP signature gene, we estimated the proportion of variance in the transcript attributable to the corresponding *cis-* or *trans-*eQTLs ($h_{eQTL}^{2}$) using the formula:

$$h_{eQTL}^{2}=max\left(0,\frac{\sigma_{eQTL.null}^{2}+\sigma_{err.null}^{2}-\sigma_{eQTL.full}^{2}-\sigma_{err.full}^{2}}{\sigma_{gene}^{2}}\right)$$

where $\sigma_{gene}^{2}$ was the total phenotypic variance of a gene expression trait; $\sigma_{eQTL.full}^{2}$ and $\sigma_{err.full}^{2}$ were the variance and the residual error, respectively, when modeling with the tested eQTL; $\sigma_{eQTL.null}^{2}$ and $\sigma_{err.null}^{2}$ were the variance and the residual error when modeling without the tested eQTL.

### Functional category enrichment analysis

In order to understand the biological themes within the global gene expression changes in relation to BP, we performed gene set enrichment analysis[29] to test for enrichment of any gene ontology (GO) biology process[30] or KEGG pathways[31]. “Metric for ranking gene” parameters were configured to the beta value of the meta-analysis, in order to look at the top enriched functions for BP associated up-regulated and down-regulated gene expression changes respectively. One thousand random permutations were conducted and the significance level was set at FDR≤ 0.25 to allow for exploratory discovery [29].

### Members of International Consortium for Blood Pressure GWAS (ICBP)

Steering Committee (alphabetical)

Gonçalo Abecasis, Murielle Bochud, Mark Caulfield (co-chair), Aravinda Chakravarti, Dan Chasman, Georg Ehret (co-chair), Paul Elliott, Andrew Johnson, Louise Wain, Martin Larson, Daniel Levy (co-chair), Patricia Munroe (co-chair), Christopher Newton-Cheh (co-chair), Paul O'Reilly, Walter Palmas, Bruce Psaty, Kenneth Rice, Albert Smith, Harold Snider, Martin Tobin, Cornelia Van Duijn, Germaine Verwoert.

Members

Georg B. Ehret^1,2,3^, Patricia B. Munroe^4^, Kenneth M. Rice^5^, Murielle Bochud^2^, Andrew D. Johnson^6,7^, Daniel I. Chasman^8,9^, Albert V. Smith^10,11^, Martin D. Tobin^12^, Germaine C. Verwoert^13,14,15^, Shih-Jen Hwang^6,16,7^, Vasyl Pihur^1^, Peter Vollenweider^17^, Paul F. O'Reilly^18^, Najaf Amin^13^, Jennifer L Bragg-Gresham^19^, Alexander Teumer^20^, Nicole L. Glazer^21^, Lenore Launer^22^, Jing Hua Zhao^23^, Yurii Aulchenko^13^, Simon Heath^24^, Siim Sõber^25^, Afshin Parsa^26^, Jian'an Luan^23^, Pankaj Arora^27^, Abbas Dehghan^13,14,15^, Feng Zhang^28^, Gavin Lucas^29^, Andrew A. Hicks^30^, Anne U. Jackson^31^, John F Peden^32^, Toshiko Tanaka^33^, Sarah H. Wild^34^, Igor Rudan^35,36^, Wilmar Igl^37^, Yuri Milaneschi^33^, Alex N. Parker^38^, Cristiano Fava^39,40^, John C. Chambers^18,41^, Ervin R. Fox^42^, Meena Kumari^43^, Min Jin Go^44^, Pim van der Harst^45^, Wen Hong Linda Kao^46^, Marketa Sjögren^39^, D. G. Vinay^47^, Myriam Alexander^48^, Yasuharu Tabara^49^, Sue Shaw-Hawkins^4^, Peter H. Whincup^50^, Yongmei Liu^51^, Gang Shi^52^, Johanna Kuusisto^53^, Bamidele Tayo^54^, Mark Seielstad^55,56^, Xueling Sim^57^, Khanh-Dung Hoang Nguyen^1^, Terho Lehtimäki^58^, Giuseppe Matullo^59,60^, Ying Wu^61^, Tom R. Gaunt^62^, N. Charlotte Onland-Moret^63,64^, Matthew N. Cooper^65^, Carl G.P. Platou^66^, Elin Org^25^, Rebecca Hardy^67^, Santosh Dahgam^68^, Jutta Palmen^69^, Veronique Vitart^70^, Peter S. Braund^71,72^, Tatiana Kuznetsova^73^, Cuno S.P.M. Uiterwaal^63^, Adebowale Adeyemo^74^, Walter Palmas^75^, Harry Campbell^35^, Barbara Ludwig^76^, Maciej Tomaszewski^71,72^, Ioanna Tzoulaki^77,78^, Nicholette D. Palmer^79^, CARDIoGRAM consortium^80^, CKDGen Consortium^80^, KidneyGen Consortium^80^, EchoGen consortium^80^, CHARGE-HF consortium^80^, Thor Aspelund^10,11^, Melissa Garcia^22^, Yen-Pei C. Chang^26^, Jeffrey R. O'Connell^26^, Nanette I. Steinle^26^, Diederick E. Grobbee^63^, Dan E. Arking^1^, Sharon L. Kardia^81^, Alanna C. Morrison^82^, Dena Hernandez^83^, Samer Najjar^84,85^, Wendy L. McArdle^86^, David Hadley^50,87^, Morris J. Brown^88^, John M. Connell^89^, Aroon D. Hingorani^90^, Ian N.M. Day^62^, Debbie A. Lawlor^62^, John P. Beilby^91,92^, Robert W. Lawrence^65^, Robert Clarke^93^, Rory Collins^93^, Jemma C Hopewell^93^, Halit Ongen^32^, Albert W. Dreisbach^42^, Yali Li^94^, J H. Young^95^, Joshua C. Bis^21^, Mika Kähönen^96^, Jorma Viikari^97^, Linda S. Adair^98^, Nanette R. Lee^99^, Ming-Huei Chen^100^, Matthias Olden^101,102^, Cristian Pattaro^30^, Judith A. Hoffman Bolton^103^, Anna Köttgen^104,103^, Sven Bergmann^105,106^, Vincent Mooser^107^, Nish Chaturvedi^108^, Timothy M. Frayling^109^, Muhammad Islam^110^, Tazeen H. Jafar^110^, Jeanette Erdmann^111^, Smita R. Kulkarni^112^, Stefan R. Bornstein^76^, Jürgen Grässler^76^, Leif Groop^113,114^, Benjamin F. Voight^115^, Johannes Kettunen^116,126^, Philip Howard^117^, Andrew Taylor^43^, Simonetta Guarrera^60^, Fulvio Ricceri^59,60^, Valur Emilsson^118^, Andrew Plump^118^, Inês Barroso^119,120^, Kay-Tee Khaw^48^, Alan B. Weder^121^, Steven C. Hunt^122^, Yan V. Sun^81^, Richard N. Bergman^123^, Francis S. Collins^124^, Lori L. Bonnycastle^124^, Laura J. Scott^31^, Heather M. Stringham^31^, Leena Peltonen^119,125,126,127^, Markus Perola^125^, Erkki Vartiainen^125^, Stefan-Martin Brand^128,129^, Jan A. Staessen^73^, Thomas J. Wang^6,130^, Paul R. Burton^12,72^, Maria Soler Artigas^12^, Yanbin Dong^131^, Harold Snieder^132,131^, Xiaoling Wang^131^, Haidong Zhu^131^, Kurt K. Lohman^133^, Megan E. Rudock^51^, Susan R Heckbert^134,135^, Nicholas L Smith^134,136,135^, Kerri L Wiggins^137^, Ayo Doumatey^74^, Daniel Shriner^74^, Gudrun Veldre^25,138^, Margus Viigimaa^139,140^, Sanjay Kinra^141^, Dorairajan Prabhakaran^142^, Vikal Tripathy^142^, Carl D. Langefeld^79^, Annika Rosengren^143^, Dag S. Thelle^144^, Anna Maria Corsi^145^, Andrew Singleton^83^, Terrence Forrester^146^, Gina Hilton^1^, Colin A. McKenzie^146^, Tunde Salako^147^, Naoharu Iwai^148^, Yoshikuni Kita^149^, Toshio Ogihara^150^, Takayoshi Ohkubo^149,151^, Tomonori Okamura^148^, Hirotsugu Ueshima^152^, Satoshi Umemura^153^, Susana Eyheramendy^154^, Thomas Meitinger^155,156^, H.-Erich Wichmann^157,158,159^, Yoon Shin Cho^44^, Hyung-Lae Kim^44^, Jong-Young Lee^44^, James Scott^160^, Joban S. Sehmi^160,41^, Weihua Zhang^18^, Bo Hedblad^39^, Peter Nilsson^39^, George Davey Smith^62^, Andrew Wong^67^, Narisu Narisu^124^, Alena Stančáková^53^, Leslie J. Raffel^161^, Jie Yao^161^, Sekar Kathiresan^162,27^, Chris O'Donnell^163,27,9^, Stephen M. Schwartz^134^, M. Arfan Ikram^13,15^, W. T. Longstreth Jr.^164^, Thomas H. Mosley^165^, Sudha Seshadri^166^, Nick R.G. Shrine^12^, Louise V. Wain^12^, Mario A. Morken^124^, Amy J. Swift^124^, Jaana Laitinen^167^, Inga Prokopenko^51,168^, Paavo Zitting^169^, Jackie A. Cooper^69^, Steve E. Humphries^69^, John Danesh^48^, Asif Rasheed^170^, Anuj Goel^32^, Anders Hamsten^171^, Hugh Watkins^32^, Stephan J.L. Bakker^172^, Wiek H. van Gilst^45^, Charles S. Janipalli^47^, K. Radha Mani^47^, Chittaranjan S. Yajnik^112^, Albert Hofman^13^, Francesco U.S. Mattace-Raso^13,14^, Ben A. Oostra^173^, Ayse Demirkan^13^, Aaron Isaacs^13^, Fernando Rivadeneira^13,14^, Edward G Lakatta^174^, Marco Orru^175,176^, Angelo Scuteri^174^, Mika Ala-Korpela^177,178,179^, Antti J Kangas^177^, Leo-Pekka Lyytikäinen^58^, Pasi Soininen^177,178^, Taru Tukiainen^180,181,177^, Peter Würtz^177,18,180^, Rick Twee-Hee Ong^56,57,182^, Marcus Dörr^183^, Heyo K. Kroemer^184^, Uwe Völker^20^, Henry Völzke^185^, Pilar Galan^186^, Serge Hercberg^186^, Mark Lathrop^24^, Diana Zelenika^24^, Panos Deloukas^119^, Massimo Mangino^28^, Tim D. Spector^28^, Guangju Zhai^28^, James F. Meschia^187^, Michael A. Nalls^83^, Pankaj Sharma^188^, Janos Terzic^189^, M. J. Kranthi Kumar^47^, Matthew Denniff^71^, Ewa Zukowska-Szczechowska^190^, Lynne E. Wagenknecht^79^, F. Gerald R. Fowkes^191^, Fadi J. Charchar^192^, Peter E.H. Schwarz^193^, Caroline Hayward^70^, Xiuqing Guo^161^, Charles Rotimi^74^, Michiel L. Bots^63^, Eva Brand^194^, Nilesh J. Samani^71,72^, Ozren Polasek^195^, Philippa J. Talmud^69^, Fredrik Nyberg^68,196^, Diana Kuh^67^, Maris Laan^25^, Kristian Hveem^66^, Lyle J. Palmer^197,198^, Yvonne T. van der Schouw^63^, Juan P. Casas^199^, Karen L. Mohlke^61^, Paolo Vineis^200,60^, Olli Raitakari^201^, Santhi K. Ganesh^202^, Tien Y. Wong^203,204^, E Shyong Tai^205,57,206^, Richard S. Cooper^54^, Markku Laakso^53^, Dabeeru C. Rao^207^, Tamara B. Harris^22^, Richard W. Morris^208^, Anna F. Dominiczak^209^, Mika Kivimaki^210^, Michael G. Marmot^210^, Tetsuro Miki^49^, Danish Saleheen^170,48^, Giriraj R. Chandak^47^, Josef Coresh^211^, Gerjan Navis^212^, Veikko Salomaa^125^, Bok-Ghee Han^44^, Xiaofeng Zhu^94^, Jaspal S. Kooner^160,41^, Olle Melander^39^, Paul M Ridker^8,213,9^, Stefania Bandinelli^214^, Ulf B. Gyllensten^37^, Alan F. Wright^70^, James F. Wilson^34^, Luigi Ferrucci^33^, Martin Farrall^32^, Jaakko Tuomilehto^215,216,217,218^, Peter P. Pramstaller^30,219^, Roberto Elosua^29,220^, Nicole Soranzo^119,28^, Eric J.G. Sijbrands^13,14^, David Altshuler^221,115^, Ruth J.F. Loos^23^, Alan R. Shuldiner^26,222^, Christian Gieger^157^, Pierre Meneton^223^, Andre G. Uitterlinden^13,14,15^, Nicholas J. Wareham^23^, Vilmundur Gudnason^10,11^, Jerome I. Rotter^161^, Rainer Rettig^224^, Manuela Uda^175^, David P. Strachan^50^, Jacqueline C.M. Witteman^13,15^, Anna-Liisa Hartikainen^225^, Jacques S. Beckmann^105,226^, Eric Boerwinkle^227^, Ramachandran S. Vasan^6,228^, Michael Boehnke^31^, Martin G. Larson^6,229^, Marjo-Riitta Järvelin^18,230,231,232,233^, Bruce M. Psaty^21,135*^, Gonçalo R Abecasis^19*^, Aravinda Chakravarti^1^, Paul Elliott^18,233*^, Cornelia M. van Duijn^13,234*^, Christopher Newton-Cheh^27,115^, Daniel Levy^6,16,7^, Mark J. Caulfield^4^, Toby Johnson^4^

Affiliations

1. Center for Complex Disease Genomics, McKusick-Nathans Institute of Genetic Medicine, Johns Hopkins University School of Medicine, Baltimore, MD 21205, USA
2. Institute of Social and Preventive Medicine (IUMSP), Centre Hospitalier Universitaire Vaudois and University of Lausanne, Bugnon 17, 1005 Lausanne, Switzerland
3. Cardiology, Department of Specialties of Internal Medicine, Geneva University Hospital, Rue Gabrielle-Perret-Gentil 4, 1211 Geneva 14, Switzerland
4. Clinical Pharmacology and The Genome Centre, William Harvey Research Institute, Barts and The London School of Medicine and Dentistry, Queen Mary University of London, London EC1M 6BQ, UK
5. Department of Biostatistics, University of Washington, Seattle, WA, USA
6. Framingham Heart Study, Framingham, MA, USA
7. National Heart Lung, and Blood Institute, Bethesda, MD, USA
8. Division of Preventive Medicine, Brigham and Women's Hospital, 900 Commonwealth Avenue East, Boston MA 02215, USA
9. Harvard Medical School, Boston, MA, USA
10. Icelandic Heart Association, Kopavogur, Iceland
11. University of Iceland, Reykajvik, Iceland
12. Department of Health Sciences, University of Leicester, University Rd, Leicester LE1 7RH, UK
13. Department of Epidemiology, Erasmus Medical Center, PO Box 2040, 3000 CA, Rotterdam, The Netherlands
14. Department of Internal Medicine, Erasmus Medical Center, Rotterdam, The Netherlands
15. Netherlands Consortium for Healthy Aging (NCHA), Netherland Genome Initiative (NGI), The Netherlands
16. Center for Population Studies, National Heart Lung, and Blood Institute, Bethesda, MD, USA
17. Department of Internal Medicine, Centre Hospitalier Universitaire Vaudois, 1011 Lausanne, Switzerland
18. Department of Epidemiology and Biostatistics, School of Public Health, Imperial College London, Norfolk Place, London W2 1PG, UK
19. Center for Statistical Genetics, Department of Biostatistics, University of Michigan School of Public Health, Ann Arbor, MI 48103, USA
20. Interfaculty Institute for Genetics and Functional Genomics, Ernst-Moritz-Arndt-University Greifswald, 17487 Greifswald, Germany
21. Cardiovascular Health Research Unit, Departments of Medicine, Epidemiology and Health Services, University of Washington, Seattle, WA, USA
22. Laboratory of Epidemiology, Demography, Biometry, National Institute on Aging, National Institutes of Health, Bethesda, Maryland 20892, USA
23. MRC Epidemiology Unit, Institute of Metabolic Science, Cambridge CB2 0QQ, UK
24. Centre National de Génotypage, Commissariat à L'Energie Atomique, Institut de Génomique, Evry, France
25. Institute of Molecular and Cell Biology, University of Tartu, Riia 23, Tartu 51010, Estonia
26. University of Maryland School of Medicine, Baltimore, MD, USA, 21201, USA
27. Center for Human Genetic Research, Cardiovascular Research Center, Massachusetts General Hospital, Boston, Massachusetts, 02114, USA
28. Department of Twin Research & Genetic Epidemiology, King's College London, UK
29. Cardiovascular Epidemiology and Genetics, Institut Municipal d'Investigacio Medica, Barcelona Biomedical Research Park, 88 Doctor Aiguader, 08003 Barcelona, Spain
30. Institute of Genetic Medicine, European Academy Bozen/Bolzano (EURAC), Viale Druso 1, 39100 Bolzano, Italy—Affiliated Institute of the University of Lübeck, Germany
31. Department of Biostatistics, Center for Statistical Genetics, University of Michigan, Ann Arbor, Michigan, 48109, USA
32. Department of Cardiovascular Medicine, The Wellcome Trust Centre for Human Genetics, University of Oxford, Oxford, OX3 7BN, UK
33. Clinical Research Branch, National Institute on Aging, Baltimore MD 21250, USA
34. Centre for Population Health Sciences, University of Edinburgh, EH89AG, UK
35. Centre for Population Health Sciences and Institute of Genetics and Molecular Medicine, College of Medicine and Vet Medicine, University of Edinburgh, EH8 9AG, UK
36. Croatian Centre for Global Health, University of Split, Croatia
37. Department of Genetics and Pathology, Rudbeck Laboratory, Uppsala University, SE-751 85 Uppsala, Sweden
38. Amgen, 1 Kendall Square, Building 100, Cambridge, MA 02139, USA
39. Department of Clinical Sciences, Lund University, Malmö, Sweden
40. Department of Medicine, University of Verona, Italy
41. Ealing Hospital, London, UB1 3HJ, UK
42. Department of Medicine, University of Mississippi Medical Center, USA
43. Genetic Epidemiology Group, Epidemiology and Public Health, UCL, London, WC1E 6BT, UK
44. Center for Genome Science, National Institute of Health, Seoul, Korea
45. Department of Cardiology, University Medical Center Groningen, University of Groningen, The Netherlands
46. Departments of Epidemiology and Medicine, Johns Hopkins University, Baltimore MD, USA
47. Centre for Cellular and Molecular Biology (CCMB), Council of Scientific and Industrial Research (CSIR), Uppal Road, Hyderabad 500 007, India
48. Department of Public Health and Primary Care, University of Cambridge, CB1 8RN, UK
49. Department of Basic Medical Research and Education, and Department of Geriatric Medicine, Ehime University Graduate School of Medicine, Toon, 791-0295, Japan
50. Division of Community Health Sciences, St George's University of London, London, SW17 0RE, UK
51. Epidemiology & Prevention, Division of Public Health Sciences, Wake Forest University School of Medicine, Winston-Salem, NC 27157, USA
52. Division of Biostatistics and Department of Genetics, School of Medicine, Washington University in St. Louis, Saint Louis, Missouri 63110, USA
53. Department of Medicine, University of Eastern Finland and Kuopio University Hospital, 70210 Kuopio, Finland
54. Department of Preventive Medicine and Epidemiology, Loyola University Medical School, Maywood, IL, USA
55. Department of Laboratory Medicine & Institute of Human Genetics, University of California San Francisco, 513 Parnassus Ave. San Francisco CA 94143, USA
56. Genome Institute of Singapore, Agency for Science, Technology and Research, Singapore, 138672, Singapore
57. Centre for Molecular Epidemiology, Yong Loo Lin School of Medicine, National University of Singapore, Singapore, 117597, Singapore
58. Department of Clinical Chemistry, University of Tampere and Tampere University Hospital, Tampere, 33521, Finland
59. Department of Genetics, Biology and Biochemistry, University of Torino, Via Santena 19, 10126, Torino, Italy
60. Human Genetics Foundation (HUGEF), Via Nizza 52, 10126, Torino, Italy
61. Department of Genetics, University of North Carolina, Chapel Hill, NC, 27599, USA
62. MRC Centre for Causal Analyses in Translational Epidemiology, School of Social & Community Medicine, University of Bristol, Bristol BS8 2BN, UK
63. Julius Center for Health Sciences and Primary Care, University Medical Center Utrecht, Heidelberglaan 100, 3508 GA Utrecht, The Netherlands
64. Complex Genetics Section, Department of Medical Genetics—DBG, University Medical Center Utrecht, 3508 GA Utrecht, The Netherlands
65. Centre for Genetic Epidemiology and Biostatistics, University of Western Australia, Crawley, WA, Australia
66. HUNT Research Centre, Department of Public Health and General Practice, Norwegian University of Science and Technology, 7600 Levanger, Norway
67. MRC Unit for Lifelong Health & Ageing, London, WC1B 5JU, UK
68. Occupational and Environmental Medicine, Department of Public Health and Community Medicine, Institute of Medicine, Sahlgrenska Academy, University of Gothenburg, 40530 Gothenburg, Sweden
69. Centre for Cardiovascular Genetics, University College London, London WC1E 6JF, UK
70. MRC Human Genetics Unit and Institute of Genetics and Molecular Medicine, Edinburgh, EH2, UK
71. Department of Cardiovascular Sciences, University of Leicester, Glenfield Hospital, Leicester, LE3 9QP, UK
72. Leicester NIHR Biomedical Research Unit in Cardiovascular Disease, Glenfield Hospital, Leicester, LE3 9QP, UK
73. Studies Coordinating Centre, Division of Hypertension and Cardiac Rehabilitation, Department of Cardiovascular Diseases, University of Leuven, Campus Sint Rafaël, Kapucijnenvoer 35, Block D, Box 7001, 3000 Leuven, Belgium
74. Center for Research on Genomics and Global Health, National Human Genome Research Institute, Bethesda, MD 20892, USA
75. Columbia University, NY, USA
76. Department of Medicine III, Medical Faculty Carl Gustav Carus at the Technical University of Dresden, 01307 Dresden, Germany
77. Epidemiology and Biostatistics, School of Public Health, Imperial College, London, W2 1PG, UK
78. Clinical and Molecular Epidemiology Unit, Department of Hygiene and Epidemiology, University of Ioannina School of Medicine, Ioannina, Greece
79. Wake Forest University Health Sciences, Winston-Salem, NC 27157, USA
80. A list of consortium members is supplied in the Supplementary Materials
81. Department of Epidemiology, School of Public Health, University of Michigan, Ann Arbor, MI 48109, USA
82. Division of Epidemiology, Human Genetics and Environmental Sciences, School of Public Health, University of Texas at Houston Health Science Center, 12 Herman Pressler, Suite 453E, Houston, TX 77030, USA
83. Laboratory of Neurogenetics, National Institute on Aging, Bethesda, MD 20892, USA
84. Laboratory of Cardiovascular Science, Intramural Research Program, National Institute on Aging, NIH, Baltimore, Maryland, USA
85. Washington Hospital Center, Division of Cardiology, Washington DC, USA
86. ALSPAC Laboratory, University of Bristol, Bristol, BS8 2BN, UK
87. Pediatric Epidemiology Center, University of South Florida, Tampa, FL, USA
88. Clinical Pharmacology Unit, University of Cambridge, Addenbrookes Hospital, Hills Road, Cambridge CB2 2QQ, UK
89. University of Dundee, Ninewells Hospital &Medical School, Dundee, DD1 9SY, UK
90. Genetic Epidemiology Group, Department of Epidemiology and Public Health, UCL, London WC1E 6BT, UK
91. Pathology and Laboratory Medicine, University of Western Australia, Crawley, WA, Australia
92. Molecular Genetics, PathWest Laboratory Medicine, Nedlands, WA, Australia
93. Clinical Trial Service Unit and Epidemiological Studies Unit, University of Oxford, Oxford, OX3 7LF, UK
94. Department of Epidemiology and Biostatistics, Case Western Reserve University, 2103 Cornell Road, Cleveland, OH 44106, USA
95. Department of Medicine, Johns Hopkins University, Baltimore, USA
96. Department of Clinical Physiology, University of Tampere and Tampere University Hospital, Tampere, 33521, Finland
97. Department of Medicine, University of Turku and Turku University Hospital, Turku, 20521, Finland
98. Department of Nutrition, University of North Carolina, Chapel Hill, NC, 27599, USA
99. Office of Population Studies Foundation, University of San Carlos, Talamban, Cebu City 6000, Philippines
100. Department of Neurology and Framingham Heart Study, Boston University School of Medicine, Boston, MA, 02118, USA
101. Department of Internal Medicine II, University Medical Center Regensburg, 93053 Regensburg, Germany
102. Department of Epidemiology and Preventive Medicine, University Medical Center Regensburg, 93053 Regensburg, Germany
103. Department of Epidemiology, Johns Hopkins University, Baltimore MD, USA
104. Renal Division, University Hospital Freiburg, Germany
105. Département de Génétique Médicale, Université de Lausanne, 1015 Lausanne, Switzerland
106. Swiss Institute of Bioinformatics, 1015 Lausanne, Switzerland
107. Division of Genetics, GlaxoSmithKline, Philadelphia, Pennsylvania 19101, USA
108. International Centre for Circulatory Health, National Heart & Lung Institute, Imperial College, London, UK
109. Genetics of Complex Traits, Peninsula Medical School, University of Exeter, UK
110. Department of Community Health Sciences & Department of Medicine, Aga Khan University, Karachi, Pakistan
111. Medizinische Klinik II, Universität zu Lübeck, Lübeck, Germany
112. Diabetes Unit, KEM Hospital and Research Centre, Rasta Peth, Pune-411011, Maharashtra, India
113. Department of Clinical Sciences, Diabetes and Endocrinology Research Unit, University Hospital, Malmö, Sweden
114. Lund University, Malmö 20502, Sweden
115. Program in Medical and Population Genetics, Broad Institute of Harvard and MIT, Cambridge, Massachusetts, 02139, USA
116. Department of Chronic Disease Prevention, National Institute for Health and Welfare, FIN-00251 Helsinki, Finland
117. William Harvey Research Institute, Barts and The London School of Medicine and Dentistry, Queen Mary University of London, London EC1M 6BQ, UK
118. Merck Research Laboratory, 126 East Lincoln Avenue, Rahway, NJ 07065, USA
119. Wellcome Trust Sanger Institute, Hinxton, CB10 1SA, UK
120. University of Cambridge Metabolic Research Labs, Institute of Metabolic Science Addenbrooke's Hospital, CB2 OQQ, Cambridge, UK
121. Division of Cardiovascular Medicine, Department of Internal Medicine, University of Michigan Medical School, Ann Arbor, MI, USA
122. Cardiovascular Genetics, University of Utah School of Medicine, Salt Lake City, UT, USA
123. Department of Physiology and Biophysics, Keck School of Medicine, University of Southern California, Los Angeles, California 90033, USA
124. National Human Genome Research Institute, National Institutes of Health, Bethesda, Maryland 20892,USA
125. National Institute for Health and Welfare, 00271 Helsinki, Finland
126. FIMM, Institute for Molecular Medicine, Finland, Biomedicum, P.O. Box 104, 00251 Helsinki, Finland
127. Broad Institute, Cambridge, Massachusetts 02142, USA
128. Leibniz-Institute for Arteriosclerosis Research, Department of Molecular Genetics of Cardiovascular Disease, University of Münster, Münster, Germany
129. Medical Faculty of the Westfalian Wilhelms University Muenster, Department of Molecular Genetics of Cardiovascular Disease, University of Muenster, Muenster, Germany
130. Division of Cardiology, Massachusetts General Hospital, Boston, MA, USA
131. Georgia Prevention Institute, Department of Pediatrics, Medical College of Georgia, Augusta, GA, USA
132. Unit of Genetic Epidemiology and Bioinformatics, Department of Epidemiology, University Medical Center Groningen, University of Groningen, Groningen, The Netherlands
133. Department of Biostatical Sciences, Division of Public Health Sciences, Wake Forest University School of Medicine, Winston-Salem, NC 27157, USA
134. Department of Epidemiology, University of Washington, Seattle, WA, 98195, USA
135. Group Health Research Institute, Group Health Cooperative, Seattle, WA, USA
136. Seattle Epidemiologic Research and Information Center, Veterans Health Administration Office of Research & Development, Seattle, WA 98108, USA
137. Department of Medicine, University of Washington, 98195, USA
138. Department of Cardiology, University of Tartu, L. Puusepa 8, 51014 Tartu, Estonia
139. Tallinn University of Technology, Institute of Biomedical Engineering, Ehitajate tee 5, 19086 Tallinn, Estonia
140. Centre of Cardiology, North Estonia Medical Centre, Sütiste tee 19, 13419 Tallinn, Estonia
141. Division of Non-communicable disease Epidemiology, The London School of Hygiene and Tropical Medicine London, Keppel Street, London WC1E 7HT, UK
142. South Asia Network for Chronic Disease, Public Health Foundation of India, C-1/52, SDA, New Delhi 100016, India
143. Department of Emergency and Cardiovascular Medicine, Institute of Medicine, Sahlgrenska Academy, University of Gothenburg, 41685 Gothenburg, Sweden
144. Department of Biostatistics, Institute of Basic Medical Sciences, University of Oslo, 0317 Oslo, Norway
145. Tuscany Regional Health Agency, Florence, Italy
146. Tropical Medicine Research Institute, University of the West Indies, Mona, Kingston, Jamaica
147. University of Ibadan, Ibadan, Nigeria
148. Department of Genomic Medicine, and Department of Preventive Cardiology, National Cerebral and Cardiovascular Research Center, Suita, 565-8565, Japan
149. Department of Health Science, Shiga University of Medical Science, Otsu, 520-2192, Japan
150. Department of Geriatric Medicine, Osaka University Graduate School of Medicine, Suita, 565-0871, Japan
151. Tohoku University Graduate School of Pharmaceutical Sciences and Medicine, Sendai, 980-8578, Japan
152. Lifestyle-related Disease Prevention Center, Shiga University of Medical Science, Otsu, 520-2192, Japan
153. Department of Medical Science and Cardiorenal Medicine, Yokohama City University School of Medicine, Yokohama, 236-0004, Japan
154. Department of Statistics, Pontificia Universidad Catolica de Chile, Vicuña Mackena 4860, Santiago, Chile
155. Institute of Human Genetics, Helmholtz Zentrum Munich, German Research Centre for Environmental Health, 85764 Neuherberg, Germany
156. Institute of Human Genetics, Klinikum rechts der Isar, Technical University of Munich, 81675 Munich, Germany
157. Institute of Epidemiology, Helmholtz Zentrum Munich, German Research Centre for Environmental Health, 85764 Neuherberg, Germany
158. Chair of Epidemiology, Institute of Medical Informatics, Biometry and Epidemiology, Ludwig-Maximilians-Universität, 81377 Munich, Germany
159. Klinikum Grosshadern, 81377 Munich, Germany
160. National Heart and Lung Institute, Imperial College London, London, UK, W12 0HS, UK
161. Medical Genetics Institute, Cedars-Sinai Medical Center, Los Angeles, CA, USA
162. Medical Population Genetics, Broad Institute of Harvard and MIT, 5 Cambridge Center, Cambridge MA 02142, USA
163. National Heart, Lung and Blood Institute and its Framingham Heart Study, 73 Mount Wayte Ave., Suite #2, Framingham, MA 01702, USA
164. Department of Neurology and Medicine, University of Washington, Seattle, USA
165. Department of Medicine (Geriatrics), University of Mississippi Medical Center, Jackson, MS, USA
166. Department of Neurology, Boston University School of Medicine, USA
167. Finnish Institute of Occupational Health, Finnish Institute of Occupational Health, Aapistie 1, 90220 Oulu, Finland
168. Wellcome Trust Centre for Human Genetics, University of Oxford, UK
169. Lapland Central Hospital, Department of Physiatrics, Box 8041, 96101 Rovaniemi, Finland
170. Center for Non-Communicable Diseases Karachi, Pakistan
171. Atherosclerosis Research Unit, Department of Medicine, Karolinska Institute, Stockholm, Sweden
172. Department of Internal Medicine, University Medical Center Groningen, University of Groningen, The Netherlands
173. Department of Medical Genetics, Erasmus Medical Center, Rotterdam, The Netherlands
174. Gerontology Research Center, National Institute on Aging, Baltimore, MD 21224, USA
175. Istituto di Neurogenetica e Neurofarmacologia, Consiglio Nazionale delle Ricerche, Cittadella Universitaria di Monserrato, Monserrato, Cagliari, Italy
176. Unita`Operativa Semplice Cardiologia, Divisione di Medicina, Presidio Ospedaliero Santa Barbara, Iglesias, Italy
177. Computational Medicine Research Group, Institute of Clinical Medicine, University of Oulu and Biocenter Oulu, 90014 University of Oulu, Oulu, Finland
178. NMR Metabonomics Laboratory, Department of Biosciences, University of Eastern Finland, 70211 Kuopio, Finland
179. Department of Internal Medicine and Biocenter Oulu, Clinical Research Center, 90014 University of Oulu, Oulu, Finland
180. Institute for Molecular Medicine Finland FIMM, 00014 University of Helsinki, Helsinki, Finland
181. Department of Biomedical Engineering and Computational Science, School of Science and Technology, Aalto University, 00076 Aalto, Espoo, Finland
182. NUS Graduate School for Integrative Sciences & Engineering (NGS) Centre for Life Sciences (CeLS), Singapore, 117456, Singapore
183. Department of Internal Medicine B, Ernst-Moritz-Arndt-University Greifswald, 17487 Greifswald, Germany
184. Institute of Pharmacology, Ernst-Moritz-Arndt-University Greifswald, 17487 Greifswald, Germany
185. Institute for Community Medicine, Ernst-Moritz-Arndt-University Greifswald, 17487 Greifswald, Germany
186. U557 Institut National de la Santé et de la Recherche Médicale, U1125 Institut National de la Recherche Agronomique, Université Paris 13, Bobigny, France
187. Department of Neurology, Mayo Clinic, Jacksonville, FL, USA
188. Imperial College Cerebrovascular Unit (ICCRU), Imperial College, London, W6 8RF, UK
189. Faculty of Medicine, University of Split, Croatia
190. Department of Internal Medicine, Diabetology, and Nephrology, Medical University of Silesia, 41-800, Zabrze, Poland
191. Public Health Sciences section, Division of Community Health Sciences, University of Edinburgh, Medical School, Teviot Place, Edinburgh, EH8 9AG, UK
192. School of Science and Engineering, University of Ballarat, 3353 Ballarat, Australia
193. Prevention and Care of Diabetes, Department of Medicine III, Medical Faculty Carl Gustav Carus at the Technical University of Dresden, 01307 Dresden, Germany
194. University Hospital Münster, Internal Medicine D, Münster, Germany
195. Department of Medical Statistics, Epidemiology and Medical Informatics, Andrija Stampar School of Public Health, University of Zagreb, Croatia
196. AstraZeneca R&D, 431 83 Mölndal, Sweden
197. Genetic Epidemiology & Biostatistics Platform, Ontario Institute for Cancer Research, Toronto
198. Samuel Lunenfeld Institute for Medical Research, University of Toronto, Canada
199. Faculty of Epidemiology and Population Health, London School of Hygiene and Tropical Medicine, UK
200. Department of Epidemiology and Public Health, Imperial College, Norfolk Place London W2 1PG, UK
201. Research Centre of Applied and Preventive Cardiovascular Medicine, University of Turku and the Department of Clinical Physiology, Turku University Hospital, Turku, 20521, Finland
202. Department of Internal Medicine, Division of Cardiovascular Medicine, University of Michigan Medical Center, Ann Arbor, Michigan, USA
203. Singapore Eye Research Institute, Singapore, 168751, Singapore
204. Department of Ophthalmology, National University of Singapore, Singapore, 119074, Singapore
205. Department of Medicine, Yong Loo Lin School of Medicine, National University of Singapore, Singapore, 119074, Singapore
206. Duke-National University of Singapore Graduate Medical School, Singapore, 169857, Singapore
207. Division of Biostatistics, Washington University School of Medicine, Saint Louis, MO, 63110, USA
208. Department of Primary Care & Population Health, UCL, London, UK, NW3 2PF, UK
209. BHF Glasgow Cardiovascular Research Centre, University of Glasgow, 126 University Place, Glasgow, G12 8TA, UK
210. Epidemiology Public Health, UCL, London, UK, WC1E 6BT, UK
211. Departments of Epidemiology, Biostatistics, and Medicine, Johns Hopkins University, Baltimore MD, USA
212. Division of Nephrology, Department of Internal Medicine, University Medical Center Groningen, University of Groningen, The Netherlands
213. Division of Cardiology, Brigham and Women's Hospital, 900 Commonwealth Avenue East, Boston MA 02215, USA
214. Geriatric Rehabilitation Unit, Azienda Sanitaria Firenze (ASF), Florence, Italy
215. National Institute for Health and Welfare, Diabetes Prevention Unit, 00271 Helsinki, Finland
216. Hjelt Institute, Department of Public Health, University of Helsinki, 00014 Helsinki, Finland
217. South Ostrobothnia Central Hospital, 60220 Seinäjoki, Finland
218. Red RECAVA Grupo RD06/0014/0015, Hospital Universitario La Paz, 28046 Madrid, Spain
219. Department of Neurology, General Central Hospital, 39100 Bolzano, Italy
220. CIBER Epidemiología y Salud Pública, 08003 Barcelona
221. Department of Medicine and Department of Genetics, Harvard Medical School, Boston, Massachusetts 02115, USA
222. Geriatric Research and Education Clinical Center, Veterans Administration Medical Center, Baltimore, MD, USA
223. U872 Institut National de la Santé et de la Recherche Médicale, Centre de Recherche des Cordeliers, Paris, France
224. Institute of Physiology, Ernst-Moritz-Arndt-University Greifswald, 17487 Greifswald, Germany
225. Institute of Clinical Medicine/Obstetrics and Gynecology, University of Oulu, Finland
226. Service of Medical Genetics, Centre Hospitalier Universitaire Vaudois, 1011 Lausanne, Switzerland
227. Human Genetics Center, 1200 Hermann Pressler, Suite E447 Houston, TX 77030, USA
228. Division of Epidemiology and Prevention, Boston University School of Medicine, Boston, MA, USA
229. Department of Mathematics, Boston University, Boston, MA, USA
230. Institute of Health Sciences, University of Oulu, BOX 5000, 90014 University of Oulu, Finland
231. Biocenter Oulu, University of Oulu, BOX 5000, 90014 University of Oulu, Finland
232. National Institute for Health and Welfare, Box 310, 90101 Oulu, Finland
233. MRC-HPA Centre for Environment and Health, School of Public Health, Imperial College London, Norfolk Place, London W2 1PG, UK
234. Centre of Medical Systems Biology (CMSB 1–2), NGI Erasmus Medical Center, Rotterdam, The Netherlands

## Supporting Information

S1 Fig Overall analysis framework. At first, we identified BP differentially expressed genes in six cohorts (FHS, EGCUT, RS, InCHIANT, KORA F4 and SHIP-TREND) respectively. Second, we conducted a meta-analysis of the Illumina cohorts (EGCUT, RS, InCHIANT, KORA F4 and SHIP-TREND). Third, for discovery and replication purpose, we replicated the BP signature genes identified in the FHS cohort in the Illumina cohorts. And in turn, we replicated the BP signature genes identified in Illumina cohorts in FHS cohort. Fourth, we conducted a meta-analysis in the six cohorts and reported the BP signature genes passing Bonferroni corrected *p*<0.05 (corrected for 7717 genes). And finally, we cross-analyzed the BP signature genes with blood eQTLs as well as with BP GWAS results to identify the BP signature genes having BP GWAS eQTLs.
(TIF)

S2 Fig Volcano plots of the meta-analysis results of differentially expressed genes of BP. A) SBP; B) DBP; C) HTN. The x-axis is the effect size (beta values) of meta-analysis and the y-axis is the −log10 transformed p values.
(TIF)

S3 Fig Coexpression of the eight genes associated in *cis* or *trans* with rs3184504 or rs653178 in the FHS. The numbers in the Heatmap indicate Pearson correlations between pairs of genes.
(TIF)

S1 Table Differentially expressed genes of BP at Bonferroni corrected p<0.05 in the FHS cohort. (XLSX)

S2 Table BP signature genes at Bonferroni corrected p<0.05 with *cis/trans* eQTLs. (XLSX)

S3 Table BP differentially expressed genes at FDR<0.2 in the meta-analysis of all six cohorts. (XLSX)

S4 Table Gene ontology enrichment analysis of BP signatures at FDR<0.2. (XLSX)

S5 Table BP signature genes at FDR<0.2 with *cis* eQTLs in ICBP GWAS. (XLSX)

S6 Table Technical covariates utilized for gene expression data normalization. (XLSX)

S1 Text Supplementary Results. (DOCX)

S2 Text Supplementary Materials and Methods. (DOCX)
