# Supplementary figures and images for: A Meta-analysis of Gene Expression Signatures of Blood Pressure and Hypertension
Source: PLoS Genet. 2015 Mar 18;11(3):e1005035. doi: 10.1371/journal.pgen.1005035 (PMC4365001; doi:10.1371/journal.pgen.1005035)

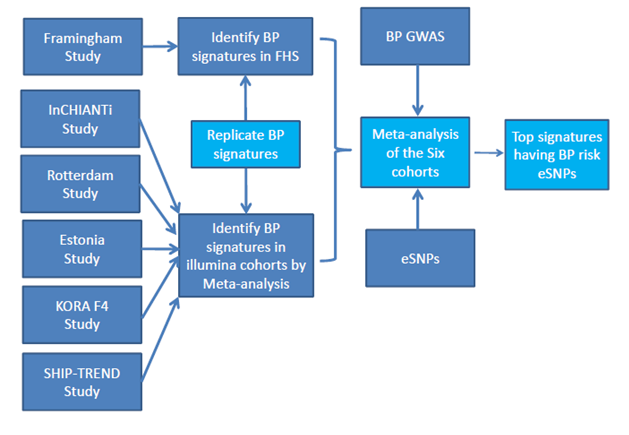

Supplement: S1 Fig — At first, we identified BP differentially expressed genes in six cohorts (FHS, EGCUT, RS, InCHIANT, KORA F4 and SHIP-TREND) respectively. Second, we conducted a meta-analysis of the Illumina cohorts (EGCUT, RS, InCHIANT, KORA F4 and SHIP-TREND). Third, for discovery and replication purpose, we replicated the BP signature genes identified in the FHS cohort in the Illumina cohorts. And in turn, we replicated the BP signature genes identified in Illumina cohorts in FHS cohort. Fourth, we conducted a meta-analysis in the six cohorts and reported the BP signature genes passing Bonferroni corrected p<0.05 (corrected for 7717 genes). And finally, we cross-analyzed the BP signature genes with blood eQTLs as well as with BP GWAS results to identify the BP signature genes having BP GWAS eQTLs. (TIF) [file pgen.1005035.s001.tif]

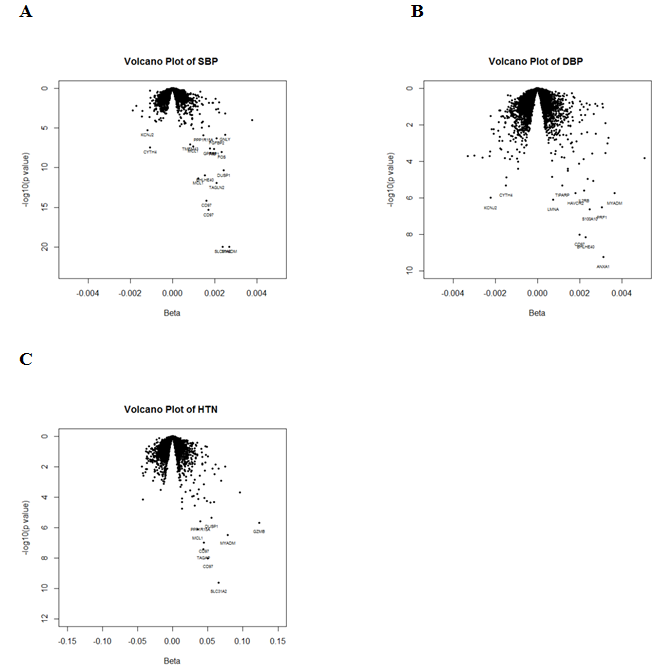

Supplement: S2 Fig — A) SBP; B) DBP; C) HTN. The x-axis is the effect size (beta values) of meta-analysis and the y-axis is the −log10 transformed p values. (TIF) [file pgen.1005035.s002.tif]

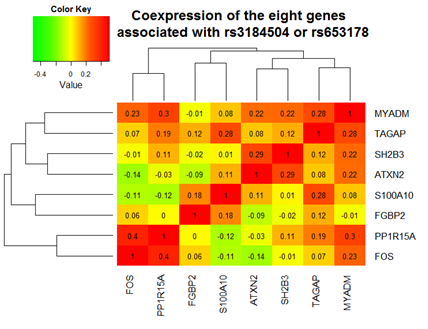

Supplement: S3 Fig — The numbers in the Heatmap indicate Pearson correlations between pairs of genes. (TIF) [file pgen.1005035.s003.tif]
