# Supplementary material for: A Meta-analysis of Gene Expression Signatures of Blood Pressure and Hypertension
Source: PLoS Genet. 2015 Mar 18;11(3):e1005035. doi: 10.1371/journal.pgen.1005035 (PMC4365001; doi:10.1371/journal.pgen.1005035)
Supplement: S1 Text — (DOCX) [file pgen.1005035.s010.docx]

**Supplementary Results**

# A Meta-analysis of Gene Expression Signatures of Blood Pressure and Hypertension

Tianxiao Huan^1,2†^, Tõnu Esko^3,4,5,6†^, Marjolein J. Peters^7,8†^, Luke C. Pilling^9†^, Katharina Schramm^10,11†^, Claudia Schurmann^12,13†^, Brian H. Chen^1,2^, Chunyu Liu^1,2^, Roby Joehanes^1,2,14,15,16^, Andrew D. Johnson^1,17^, Chen Yao^1,2^, Sai-xia Ying^14^, Paul Courchesne^1,2^, Lili Milani^3^, Nalini Raghavachari^18^, Richard Wang^19^, Poching Liu^19^, Eva Reinmaa^3^, Abbas Dehghan^8,20^, Albert Hofman^8,20^, André G. Uitterlinden^7,8,20^, Dena G. Hernandez^21^, Stefania Bandinelli^22^, Andrew Singleton^21^, David Melzer^9^, Andres Metspalu^3^, Maren Carstensen^23,24^, Harald Grallert^25,26,27^, Christian Herder^23,24^, Thomas Meitinger^10,11,28^ , Annette Peters^26,27,28^, Michael Roden^23,24,29^, Melanie Waldenberger^25,26^, Marcus Dörr^30,31^, Stephan B. Felix^30,31^, Tanja Zeller^32,33^, International Consortium for Blood Pressure GWAS (ICBP), Ramachandran Vasan^1^, Christopher J. O'Donnell^1,2^, Peter J. Munson^14^, Xia Yang^34*^, Holger Prokisch^10,11*^, Uwe Völker^12,31*^, Joyce B.J. van Meurs^7,8*^, Luigi Ferrucci^32*^, Daniel Levy ^1,2*^

^1^The National Heart, Lung, and Blood Institute's Framingham Heart Study, 73 Mt. Wayte Avenue, Framingham, MA 01702, USA; ^2^The Population Sciences Branch, Division of Intramural Research, National Heart, Lung, and Blood Institute, Bethesda, MD, USA; ^3^Estonian Genome Center, University of Tartu, Riia 23, Tartu, 51010, Estonia; ^4^Division of Endocrinology, Children’s Hospital Boston, 300 Longwood Ave, Boston, MA 02115, USA; ^5^Department of Genetics, Harvard Medical School, 25 Shattuck St, Boston, MA 02115, USA; ^6^Broad Institute of Harvard and MIT, 7 Cambridge Center, Cambridge, MA 02142, USA; ^7^Department of Internal Medicine, Erasmus Medical Centre Rotterdam, Rotterdam, The Netherlands; ^8^Netherlands Genomics Initiative–sponsored Netherlands Consortium for Healthy Aging (NGI‐NCHA), Leiden and Rotterdam, The Netherlands; ^9^ Epidemiology and Public Health Group, Medical School, University of Exeter, EX2 5DW, U.K; ^10^Institute of Human Genetics, Helmholtz Zentrum München–German Research Center for Environmental Health, Neuherberg, Germany; ^11^Institute of Human Genetics, Technische Universität München, München, Germany; ^12^Department of Functional Genomics, Interfaculty Institute for Genetics and Functional Genomics, University Medicine Greifswald, 17475 Greifswald, Germany; ^13^The Charles Bronfman Institute for Personalized Medicine, Genetics of Obesity & Related Metabolic Traits Program, Icahn School of Medicine at Mount Sinai, One Gustave L. Levy Place, New York, NY 10029, USA; ^14^Mathematical and Statistical Computing Laboratory, Center for Information Technology, National Institutes of Health, USA; ^15^Harvard Medical School, Boston, MA, USA; ^16^Hebrew SeniorLife, Boston, MA, USA; ^17^Cardiovascular Epidemiology and Human Genomics Branch, Division of Intramural Research, National Heart, Lung and Blood Institute, Bethesda, MD, USA; ^18^Division of Geriatrics and Clinical Gerontology National Institute on Aging, Bethesda MD, 20892, USA; ^19^Genomics Core facility Genetics & Developmental  Biology Center, NHLBI, USA*;* ^20^Department of Epidemiology, Erasmus Medical Centre Rotterdam, Rotterdam, The Netherland; ^21^Laboratory of Neurogenetics, National Institute on Aging, Bethesda, MD 20892; ^22^Geriatric Unit, Azienda Sanitaria Firenze, Florence, Italy, 50125; ^23^Institute for Clinical Diabetology, German Diabetes Center, Leibniz Center for Diabetes Research at Heinrich Heine University Düsseldorf, Düsseldorf, Germany; ^24^German Center for Diabetes Research (DZD e.V.), Partner Düsseldorf, Germany; ^25^Research Unit of Molecular Epidemiology, Helmholtz Zentrum München–German Research Center for Environmental Health, Neuherberg, Germany; ^26^Institute of Epidemiology II, Helmholtz Zentrum München – German Research Center for Environmental Health, Neuherberg, Germany; ^27^German Center for Diabetes Research (DZD e.V.), Partner Munich, Germany; ^28^DZHK (German Centre for Cardiovascular Research),, partner site Munich Heart Alliance, Munich, Germany; ^29^Division of Endocrinology and Diabetology, Medical Faculty, Heinrich-Heine University Düsseldorf, Düsseldorf, Germany; ^30^‎‎University Medicine Greifswald, Department of Internal Medicine B - Cardiology, 17475 Greifswald, Germany; ^31^DZHK (German Center for Cardiovascular Research), partner site Greifswald, 17475 Greifswald, Germany; ^32^Universitäres Herzzentrum Hamburg, Hamburg, Germany; ^33^DZHK (German Centre for Cardiovascular Research), partner site Hamburg/Kiel/Lübeck, Hamburg, Germany; ^34^Department of Integrative Biology and Physiology, University of California, Los Angeles, Los Angeles, CA 90095, USA; ^35^Intramural Research Program, National Institute on Aging, National Institutes of Health, Baltimore, Maryland 21224, USA.

**Short title:** *Gene expression signatures of blood pressure*

**Keywords:** Transcriptome, hypertension, blood pressure, gene expression, genetics

^†^ **These authors contribute equally.**

***Correspondence should be addressed to:**

Daniel Levy, MD

Framingham Heart Study

Population Sciences Branch

National Heart, Lung, and Blood Institute

73 Mt. Wayte Avenue, Suite 2

Framingham, MA 01702

Email: [Levyd@nih.gov](mailto:Levyd@nih.gov)

Phone: 508-935-3458

Fax: 508-872-2678

Luigi Ferrucci, M.D., PhD

Intramural Research Program,

National Institute on Aging,

National Institutes of Health,

Baltimore, Maryland 21224
Email: [ferruccilu@mail.nih.gov](mailto:ferruccilu@mail.nih.gov)

Phone 410-350-3936

Joyce B.J. van Meurs, PhD

The Rotterdam Study
Erasmus MC

Genetic Laboratory Department of Internal Medicine; room Ee579b

PO Box 2040

3000 CA, Rotterdam, the Netherlands

Email: j.vanmeurs@erasmusmc.nl
Phone: +31107038425

Holger Prokisch, PhD

Institute of Human Genetics

Helmholtz Zentrum München

Ingolstädter Landstraße 1

85764 Neuherberg, Germany

Email: prokisch@helmholtz-muenchen.de

Phone: +498931872890

Uwe Völker, PhD

Interfaculty Institute for Genetics and Functional Genomics

University Medicine Greifswald

Friedrich-Ludwig-Jahn-Str. 15A

17475 Greifswald, Germany

Email: voelker@uni-greifswald.de

Phone: +49-3834-865870

Xia Yang, PhD

Department of Integrative Biology and Physiology

University of California, Los Angeles

Los Angeles, CA 90095

Email: [xyang123@ucla.edu](mailto:xyang123@ucla.edu)

Phone: 310-206-1812

Fax:  310-206-9184

**Gene ontology enrichment analysis of BP signature genes at FDR<0.2**

The 34 BP signature genes from the meta-analysis of the six studies (at Bonferroni corrected p<0.05, reported in the main text) did not show any enrichment using gene ontology (GO) - biological process categories, possibly because the number of genes was small. In order to better understand the biological themes within the data, we liberalized the threshold for selecting differentially expressed genes in the meta-analysis results by using a FDR threshold of 0.2. This resulted in the identification of 224 unique genes (142 for SBP, 137 for DBP, and 45 for HTN, **Table S3**). Of the 224 unique genes, 109 were positively correlated with BP phenotypes and 115 were negatively correlated. The GO analysis of this larger BP gene set showed enrichment for inflammatory response (*p*=2.7e-9), chemotaxis (*p*=3.9e-6), and positive regulation of apoptosis (*p*=5.0e-6). The positively correlated gene set showed enrichment for positive regulation of apoptosis (*p*=2.0e-6), and the negatively correlated set showed enrichment for inflammatory response (*p*=1.8e-7) (**Table S4**). The biological processes suggested by GO analysis are consistent with the results suggested by GSEA (reported in the main text).

**Genetic effects on expression of BP signature genes at FDR<0.2**

We did not find any BP GWAS SNPs that were *cis-* associated with the 34 top BP signature genes from the meta-analysis (at Bonferroni corrected *p*<0.05), we further checked if any BP GWAS SNPs were *cis-* associated with the 224 BP signature genes at FDR<0.2. For every gene, we retrieved its peak *cis-*eSNP with the lowest p value for association with BP in the ICBP BP GWAS [main_text: Ref. 3] (**Table S5**). One signature gene (CSK) harbored a *cis-*eSNP (rs1378942) associated in GWAS with BP at *p*<5e-8 and 10 signature genes (*APBB3, RNF10, AHNAK, EIF2C2, ACAP1, ACSS2, TSPAN5, CREB1, AKAP13,* and *DPYSL2*) had *cis-*eSNPs associated with BP at 5e-8≤*p*<1e-3.

As described in the main text, we found 6 genes *trans-* associated with a BP GWAS SNP (i.e., rs3184504). Besides the 6 genes, we did not find any other BP signature genes at FDR<0.2 *trans-* associated with BP GWAS SNPs reported in NHGRI GWAS Catalog [main_text: Ref. 14].
