# Supplementary material for: A Meta-analysis of Gene Expression Signatures of Blood Pressure and Hypertension
Source: PLoS Genet. 2015 Mar 18;11(3):e1005035. doi: 10.1371/journal.pgen.1005035 (PMC4365001; doi:10.1371/journal.pgen.1005035)
Supplement: S2 Text — (DOCX) [file pgen.1005035.s011.docx]

**Supplementary Materials and Methods**

# A Meta-analysis of Gene Expression Signatures of Blood Pressure and Hypertension

Tianxiao Huan^1,2†^, Tõnu Esko^3,4,5,6†^, Marjolein J. Peters^7,8†^, Luke C. Pilling^9†^, Katharina Schramm^10,11†^, Claudia Schurmann^12,13†^, Brian H. Chen^1,2^, Chunyu Liu^1,2^, Roby Joehanes^1,2,14,15,16^, Andrew D. Johnson^1,17^, Chen Yao^1,2^, Sai-xia Ying^14^, Paul Courchesne^1,2^, Lili Milani^3^, Nalini Raghavachari^18^, Richard Wang^19^, Poching Liu^19^, Eva Reinmaa^3^, Abbas Dehghan^8,20^, Albert Hofman^8,20^, André G. Uitterlinden^7,8,20^, Dena G. Hernandez^21^, Stefania Bandinelli^22^, Andrew Singleton^21^, David Melzer^9^, Andres Metspalu^3^, Maren Carstensen^23,24^, Harald Grallert^25,26,27^, Christian Herder^23,24^, Thomas Meitinger^10,11,28^ , Annette Peters^26,27,28^, Michael Roden^23,24,29^, Melanie Waldenberger^25,26^, Marcus Dörr^30,31^, Stephan B. Felix^30,31^, Tanja Zeller^32,33^, International Consortium for Blood Pressure GWAS (ICBP), Ramachandran Vasan^1^, Christopher J. O'Donnell^1,2^, Peter J. Munson^14^, Xia Yang^34*^, Holger Prokisch^10,11*^, Uwe Völker^12,31*^, Joyce B.J. van Meurs^7,8*^, Luigi Ferrucci^32*^, Daniel Levy ^1,2*^

^1^The National Heart, Lung, and Blood Institute's Framingham Heart Study, 73 Mt. Wayte Avenue, Framingham, MA 01702, USA; ^2^The Population Sciences Branch, Division of Intramural Research, National Heart, Lung, and Blood Institute, Bethesda, MD, USA; ^3^Estonian Genome Center, University of Tartu, Riia 23, Tartu, 51010, Estonia; ^4^Division of Endocrinology, Children’s Hospital Boston, 300 Longwood Ave, Boston, MA 02115, USA; ^5^Department of Genetics, Harvard Medical School, 25 Shattuck St, Boston, MA 02115, USA; ^6^Broad Institute of Harvard and MIT, 7 Cambridge Center, Cambridge, MA 02142, USA; ^7^Department of Internal Medicine, Erasmus Medical Centre Rotterdam, Rotterdam, The Netherlands; ^8^Netherlands Genomics Initiative–sponsored Netherlands Consortium for Healthy Aging (NGI‐NCHA), Leiden and Rotterdam, The Netherlands; ^9^ Epidemiology and Public Health Group, Medical School, University of Exeter, EX2 5DW, U.K; ^10^Institute of Human Genetics, Helmholtz Zentrum München–German Research Center for Environmental Health, Neuherberg, Germany; ^11^Institute of Human Genetics, Technische Universität München, München, Germany; ^12^Department of Functional Genomics, Interfaculty Institute for Genetics and Functional Genomics, University Medicine Greifswald, 17475 Greifswald, Germany; ^13^The Charles Bronfman Institute for Personalized Medicine, Genetics of Obesity & Related Metabolic Traits Program, Icahn School of Medicine at Mount Sinai, One Gustave L. Levy Place, New York, NY 10029, USA; ^14^Mathematical and Statistical Computing Laboratory, Center for Information Technology, National Institutes of Health, USA; ^15^Harvard Medical School, Boston, MA, USA; ^16^Hebrew SeniorLife, Boston, MA, USA; ^17^Cardiovascular Epidemiology and Human Genomics Branch, Division of Intramural Research, National Heart, Lung and Blood Institute, Bethesda, MD, USA; ^18^Division of Geriatrics and Clinical Gerontology National Institute on Aging, Bethesda MD, 20892, USA; ^19^Genomics Core facility Genetics & Developmental  Biology Center, NHLBI, USA*;* ^20^Department of Epidemiology, Erasmus Medical Centre Rotterdam, Rotterdam, The Netherland; ^21^Laboratory of Neurogenetics, National Institute on Aging, Bethesda, MD 20892; ^22^Geriatric Unit, Azienda Sanitaria Firenze, Florence, Italy, 50125; ^23^Institute for Clinical Diabetology, German Diabetes Center, Leibniz Center for Diabetes Research at Heinrich Heine University Düsseldorf, Düsseldorf, Germany; ^24^German Center for Diabetes Research (DZD e.V.), Partner Düsseldorf, Germany; ^25^Research Unit of Molecular Epidemiology, Helmholtz Zentrum München–German Research Center for Environmental Health, Neuherberg, Germany; ^26^Institute of Epidemiology II, Helmholtz Zentrum München – German Research Center for Environmental Health, Neuherberg, Germany; ^27^German Center for Diabetes Research (DZD e.V.), Partner Munich, Germany; ^28^DZHK (German Centre for Cardiovascular Research),, partner site Munich Heart Alliance, Munich, Germany; ^29^Division of Endocrinology and Diabetology, Medical Faculty, Heinrich-Heine University Düsseldorf, Düsseldorf, Germany; ^30^‎‎University Medicine Greifswald, Department of Internal Medicine B - Cardiology, 17475 Greifswald, Germany; ^31^DZHK (German Center for Cardiovascular Research), partner site Greifswald, 17475 Greifswald, Germany; ^32^Universitäres Herzzentrum Hamburg, Hamburg, Germany; ^33^DZHK (German Centre for Cardiovascular Research), partner site Hamburg/Kiel/Lübeck, Hamburg, Germany; ^34^Department of Integrative Biology and Physiology, University of California, Los Angeles, Los Angeles, CA 90095, USA; ^35^Intramural Research Program, National Institute on Aging, National Institutes of Health, Baltimore, Maryland 21224, USA.

**Short title:** *Gene expression signatures of blood pressure*

**Keywords:** Transcriptome, hypertension, blood pressure, gene expression, genetics

^†^ **These authors contribute equally.**

***Correspondence should be addressed to:**

Daniel Levy, MD

Framingham Heart Study

Population Sciences Branch

National Heart, Lung, and Blood Institute

73 Mt. Wayte Avenue, Suite 2

Framingham, MA 01702

Email: [Levyd@nih.gov](mailto:Levyd@nih.gov)

Phone: 508-935-3458

Fax: 508-872-2678

Luigi Ferrucci, M.D., PhD

Intramural Research Program,

National Institute on Aging,

National Institutes of Health,

Baltimore, Maryland 21224
Email: [ferruccilu@mail.nih.gov](mailto:ferruccilu@mail.nih.gov)

Phone 410-350-3936

Joyce B.J. van Meurs, PhD

The Rotterdam Study
Erasmus MC

Genetic Laboratory Department of Internal Medicine; room Ee579b

PO Box 2040

3000 CA, Rotterdam, the Netherlands

Email: j.vanmeurs@erasmusmc.nl
Phone: +31107038425

Holger Prokisch, PhD

Institute of Human Genetics

Helmholtz Zentrum München

Ingolstädter Landstraße 1

85764 Neuherberg, Germany

Email: prokisch@helmholtz-muenchen.de

Phone: +498931872890

Uwe Völker, PhD

Interfaculty Institute for Genetics and Functional Genomics

University Medicine Greifswald

Friedrich-Ludwig-Jahn-Str. 15A

17475 Greifswald, Germany

Email: voelker@uni-greifswald.de

Phone: +49-3834-865870

Xia Yang, PhD

Department of Integrative Biology and Physiology

University of California, Los Angeles

Los Angeles, CA 90095

Email: [xyang123@ucla.edu](mailto:xyang123@ucla.edu)

Phone: 310-206-1812

Fax:  310-206-9184

**FHS**

Biosamples collected from all available Framingham Heart Study (FHS) participants in Offspring cohort participants who attended their eighth clinic exam (2005-2008) and Third generation cohort participants who attended their second exam (2008-2011). Among the total 5726 samples in FHS, 3679 individuals who were not receiving antihypertensive treatment were chosen for this study.

Whole blood samples (2.5ml) were collected in PAXgene™ tubes (PreAnalytiX, Hombrechtikon, Switzerland). Expression profiling was carried out on samples that passed RNA quality control. RNA expression was conducted using the Affymetrix Human Exon Array ST 1.0 (Affymetrix, Inc., Santa Clara, CA). The core probe sets were annotated using the Affymetrix annotation file from Netaffx (www.netaffx.com, HuEx-1_0-st-v2.na29.hg18.probeset.csv). All data used herein are available online in dbGaP (http://www.ncbi.nlm.nih.gov/gap; accession number phs000007). Details of the design, sampling, RNA isolation, and mRNA measurement were previously described in [[1](#_ENREF_1),[2](#_ENREF_2)].

The cell counts proportions of whole blood were measured in 2138 FHS individuals. We estimated the cell counts proportions in the remaining samples by Partial Least Squares regression [[3](#_ENREF_3)]. The estimated cell counts proportion values were highly consistent with the measured cell counts proportion.

First, raw gene expression levels were quartile normalized and log2-transformed. Second, a linear mixed model was used to adjust gene expression values for cell counts (white blood cells, red blood cells, lymphocytes, neutrophils, platelets, monocytes and eosinophils), technical covariates and the first principal component, by utilizing the *lmer()* R function, and the residuals (gene_resid) were retained. **Table S6** lists the 11 technical covariates; batch was modeled as a random effect and all the other covariates as fixed effects. A linear mixed model was used to examine the association of blood pressure phenotypes (SBP, DBP, and HTN as explanatory variables) and gene_resid (dependent variables) accounting for age, sex, and BMI as fixed effects and familial relatedness as a random effect, implemented in *lmekin()* R function [[4](#_ENREF_4)].

**EGCUT**

The Estonian Gene Expression Cohort [8] is drawn from the population-based Biobank of the Estonian Genome Center of the University of Tartu (EGCUT). The whole project is conducted according to the Estonian Genes Research Act and all participants have signed the informed consent. The current cohort size is over 51,515 participants (collected between 2003 to 2010), from 18 years of age and up, which closely reflects the age distribution of the adult Estonian population. Each participant filled out a Computer Assisted Personal interview during 1-2 hours at a recruitment office, including personal data, genealogical data, lifestyle data and medical history (diseases recorded in ICD-10 systems; the use of medicaments). Individuals who reported usage of diuretics, β blockers, calcium channel blockers, or angiotensin-converting-enzyme inhibitors were excluded from current study. Height, weight and blood pressure phenotypes (SBP and DBP) were measured at the recruitment office according to standard medical procedures. Cell counts were assessed from fresh blood at the United Laboratories, Tartu University Hospital, Tartu, Estonia.

Whole peripheral blood RNA samples were collected using Tempus Blood RNA Tubes (Life Technologies, NY, USA), and RNA was extracted using the Tempus Spin RNA Isolation Kit (Life Technologies, NY, USA). The quality and quantity of the RNA samples were measured using the NanoDrop 1000 Spectrophotometer (Thermo Fisher Scientific, DE, USA) and the Agilent 2100 Bioanalyzer (Agilent Technologies, CA, USA). Whole-Genome gene-expression levels were obtained by Illumina Human HT12v3 arrays (Illumina Inc, San Diego, US) according to the manufacturer’s protocols.

Raw gene expression levels were quartile normalized and log2-transformed. A least squares linear regression model ( *lm()* R function) was used to examine the association of the blood pressure phenotypes (SBP, DBP and HTN) and the gene expression levels, adjusting for age, sex, BMI, RNA quality score (RIN), batch (as factors) and cell counts. Gene expression levels were included as dependent variables, blood pressure phenotypes were used as explanatory variables, and the adjustments as covaraites.

**RS**

The Rotterdam Study (RS) ([www.epib.nl/rotterdamstudy](http://www.epib.nl/rotterdamstudy)*)* is a prospective, population-based cohort study in the district of Rotterdam, the Netherlands, and has been described in detail [[5](#_ENREF_5)]. The initial design of the study is straight-forward: a prospective cohort study among 7983 persons living in the well-defined Ommoord district in the city of Rotterdam (78% of 10,215 invitees), called Rotterdam Study I (or RS-I). They were all 55 years of age or older and the oldest participant at the start was 106 years. The study started in the second half of 1989. In 1999, 3,011 participants (out of 4472 invitees) who had become 55 years of age or moved into the study district since the start of the study were added to the cohort, called Rotterdam Study II (or RS-II). In 2006, a further extension of the cohort was initiated in which 3,932 subjects were included, aged 45 years and older (out of 6057 invited), called Rotterdam Study III (RS-III). The participants were all examined in some detail at baseline. They were interviewed at home and then had an extensive set of examinations in a specially built research facility in the centre of their district. These examinations were repeated every 3–4 years in characteristics that could change over time. The participants in the Rotterdam Study are followed for a variety of diseases that are frequent in the elderly. Informed consent was obtained from each participant, and the medical ethics committee of the Erasmus Medical Center Rotterdam approved the study.

For the expression analyses, the RS-III cohort was used: whole-blood was collected (PAXgene Tubes – Becton Dickinson) and total RNA was isolated (PAXgene Blood RNA kits - Qiagen). To ensure a constant high quality of the RNA preparations, all RNA samples were analyzed using the Labchip GX (Calliper) according to the manufacturer’s instructions. Samples with an RNA Quality Score > 7 were amplified and labelled (Ambion TotalPrep RNA), and hybridized to the Illumina HumanHT12v4 Expression Beadchips as described by the manufacturer’s protocol. Processing of the Rotterdam Study RNA samples was performed at the Genetic Laboratory of Internal Medicine, Erasmus University Medical Center Rotterdam. The RS-III expression dataset is available at GEO (Gene Expression Omnibus) public repository under the accession GSE33828: 881 samples are available for analysis.

Two blood pressure measurements were taken with a random-zero sphygmomanometer after 5 minutes of rest with the subject in a sitting position. The mean of the 2 blood pressure values was used in the analyses. Hypertension was defined as a minimal level of 140/100 mm Hg (according to European Society of Cardiology criteria). The latter was assessed through automated linkage to pharmacies with computerized records. Assessment of antihypertensive drug usage included usage of diuretics, β blockers, calcium channel blockers, or angiotensin-converting-enzyme inhibitors at date of the blood pressure measurement. Cell counts were assessed with a Coulter® Ac-T diff2TM Hematology Analyzer at the research centre in Ommoord.

Raw gene expression levels were quartile normalized and log2-transformed. A least squares linear regression model ( *lm()* R function) was used to examine the association of the blood pressure phenotypes (SBP, DBP and HTN) and the gene expression levels, adjusting for age, sex, BMI, RNA Quality Score (RQS), plate ID (batch, as factors), and cell counts. Gene expression levels were included as dependent variables, blood pressure phenotypes were used as explanatory variables, and the adjustments as covariates.

**InCHIANTI**

Participants attending the fourth examination (2007-2008) of the InCHIANTI cohort had their peripheral gene expression levels measured and blood pressure readings taken. After exclusions (missing data, antihypertensive treatment), 597 participants were eligible for the current study. Blood samples were collected using PAXgene™ tubes (PreAnalytiX, Hombrechtikon, Switzerland) and RNA was extracted using the PAXgene Blood mRNA kit (Qiagen, Crawley, UK) according to the manufacturer’s instructions. Whole genome expression profiling of the samples was conducted using the Illumina Human HT-12 microarray (Illumina, San Diego, USA), as previously described [[6](#_ENREF_6)].

Raw gene expression levels were quartile normalized and log2-transformed. The gene expression values were then adjusted for both hybridization and amplification batch (as factors), and cell counts prior to analysis by taking the residuals of a linear regression model (*lm()* R function), as previously described [[6](#_ENREF_6)]. Each adjusted probe was then regressed against the outcome, adjusted for the age, sex, and BMI, implemented in *lm()* R function.

**KORA F4**

KORA F4 (Cooperative Heath Research in the Region of Augsburg) is a population-based survey in the region of Augsburg in Southern Germany which was performed between 2006 and 2008. KORA exists since 1996 in the region of Augsburg in the southwest of Germany, and it is a regional research platform for population-based surveys and follow-up studies. Four cross-sectional health surveys have been performed in five-year intervals, each containing independent random samples of individuals with German nationality resident in Augsburg city or one of sixteen communities from the adjacent counties (Holle et al., 2005). The study followed the recommendations of the Declaration of Helsinki and was approved by the local ethical committees. For the expression analysis, 1002 elderly individuals aged 61 to 82 years who had already participated in the baseline survey KORA S4 (1999-2001) were used (Rathmann et al., 2009, PMID: 20002472). Gene expression profiling was performed using the Illumina HumanHT12v3 BeadChip as described elsewhere (Mehta et al., 2012, PMID: 22692066). RNA was isolated from whole blood under fasting conditions using PAXgene Blood miRNA Kit (Qiagen, Hilden, Germany). Purity and integrity of the RNA was analyzed using the Agilent Bioanalyzer with the 6000 Nano LabChip reagent set (Agilent Technologies, Germany). Samples with low quality were excluded after manually inspection. Using the Illumina TotalPrep-96 RNA Amp Kit (Ambion), 500ng of RNA was reverse transcribed into cRNA, and biotin-UTP-labeled. 3000ng of cRNA was hybridized to the Illumina HumanHT12v3 Expression BeadChips, followed by washing steps as described in the Illumina protocol. After quality control, 993 samples were available for analysis and were quantile normalized and log2 transformed.

The Hawksley random-zero sphygmomanometer was used for blood pressure (BP) measurement. Three BP recordings were taken after resting in a sitting position for about 30 minutes. BP was measured under strictly standardized conditions following the World Health Organization MONICA protocol and the American Heart Organization guidelines[[7](#_ENREF_7),[8](#_ENREF_8)]. For the present analysis, the results of the second and third measurement were averaged. Samples receiving anit-hypertensive treatment were excluded from the analysis resulting in 565 samples for all further analyses.

Raw gene expression levels were quartile normalized and log2-transformed. The gene expression values were adjusted for technical covariates (RIN, batch and sample storage time) and cell counts (white blood cells, red blood cells and platelets) prior to analysis by taking the residuals of a linear regression model (*lm()* R function). Each adjusted probe was then regressed against the outcome, adjusted for the age, sex, and BMI, implemented in *lm()* R function.

**SHIP-TREND**

The Study of Health in Pomerania (SHIP) is a longitudinal population-based cohort study in West Pomerania, a region in the northeast of Germany, assessing the prevalence and incidence of common population-relevant diseases and their risk factors. Baseline examinations for SHIP-TREND were carried out between 2008 and 2012, comprising 4420 participants. Study design and sampling methods were previously described [main_text, Ref. 10]. The present project is based on a subset of 600 subjects aged 20 to 81 years of the SHIP-TREND study population with available phenotype and gene expression data. Weight and height were measured according to written standardized instructions in accordance with the World Health Organization standards (WHO 1987). The body mass index (BMI) was calculated as weight in kg divided by the square of height in meters. After a 5 minutes rest period, systolic and diastolic blood pressures were measured three times at the right arm of seated participants using a digital blood pressure monitor (HEM-705CP, Omron Corporation, Tokyo, Japan) with a 3 minutes interval between consecutive measurements. The mean of the second and third measurements was used for the present analyses. Hypertension was defined as systolic or diastolic blood pressure of ≥140 mmHg or ≥90 mmHg, respectively. Individuals receiving anti-hypertensive medication were excluded from analysis. Blood cell counts were measured using the automated hematology analysers Sysmex XE-5000/XT-2000 (Sysmex Corporation, Kobe, Japan). All assays were performed according to the manufacturers’ recommendations by skilled technical personnel. Blood sample collection as well as RNA preparation were described in detail elsewhere [main_text, Ref. 9]. Briefly, fasting whole-blood samples were collected and stored in PAXgene Blood RNA Tubes (BD). RNA was prepared using the PAXgeneTM Blood miRNA Kit (QIAGEN, Hilden, Germany). Purity and concentration of RNA were determined using a NanoDrop ND-1000 UV-Vis Spectrophotometer (Thermo Scientific). Samples exhibiting an RNA integrity number (RIN) less than seven were excluded from further analysis. Whole genome expression profiling of the samples was conducted using the Illumina HumanHT-12 v3 BeadChip array.

Raw gene expression levels were quartile normalized and log2-transformed. A least squares linear regression model (*lm()* R function) was used to examine the association of the blood pressure phenotypes (SBP, DBP and HTN) and the gene expression levels, adjusting for age, sex, BMI, plate layout after RNA amplification (96 well plates), sample storage time (time between blood donation and RNA preparation), and cell counts. Gene expression levels were included as dependent variables, blood pressure phenotypes were used as explanatory variables, and the adjustments as covariates. A detailed workflow for the analysis of Illumina gene expression microarray data was described recently within the MetaXpress consortium [main_text, Ref. 9].

**Identification of *cis-* and *trans-* eQTLs in the FHS**

***Genotyping and quality control:*** Genotyping for all available FHS individuals (n=9274) were carried out by utilizing Affymetrix 550K and MIPS 50K platforms. A total of 503,551 SNPs with successful call rate >0.95 and Hardy-Weinberg Equilibrium (HWE) *P*>10-6 were retained. Imputation of ~36.3 million SNPs in 1000 Genomes Phase 1 SNP data was conducted using MACH. In this eQTL study, we used the 1000-genome resource imputed SNPs with minor allele frequency (MAF) >0.01 and imputation ratio >0.3, yielding approximately 8 million SNPs for analysis.

***Population stratification:*** To assess population stratification in the gene expression data, the top 10 principal components (PCs) were generated using EIGENSTRAT [[9](#_ENREF_9)] derived from genotype data. By correlating the gene expression levels with each PC, we found only 64 genes that were significantly associated with one of the ten PCs (Bonferroni corrected *p*<0.05), suggesting that population substructure has very small confounding effects in our eQTL study. For these 64 genes we adjusted for the 10 PCs in the eQTL analysis.

***eQTL identification:*** The PEER package in R was used to compute the hidden global determinants of gene expression, by modeling age, sex, and technical factors as covariates[[10](#_ENREF_10)]. Pedigree-based linear mixed regression models were used to determine the association between gene expression (adjusted for age, sex, technical covariates, familiar relatedness, and the top 20 PEER factors) and the imputed SNP genotypes from the 1000-genome resource. A *cis-*eQTL was defined as a SNP residing on the same chromosome as its associated transcript and within 1 megabase (MB) of the transcriptional start site (TSS) of the gene. The other eQTLs were defined as *trans-*eQTLs. Genomic coordinates were based on NCBI human reference genome build 37/hg19. The Benjamini-Hochberg method was used to compute FDR for *cis-* and *trans-*eQTLs respectively.

***Summary of eQTLs results:*** This analysis (the focus of another manuscript) generated 2,206,283 *cis-* eQTLs SNPs for 10,327 genes and 160,183 *trans-*eQTLs SNPs for 5,271 genes at FDR<0.05. Among the eQTLs, 69% of the top 1000 *cis-*eQTLs and 49% of the top 1000 *trans-*eQTLs were replicated in published studies.

**Gene ontology enrichment analysis**

The differentially expressed BP genes at FDR<0.2 were separated into positively correlated and inversely correlated sets (i.e. genes that are up- and down-regulated, respectively in relation to higher BP levels). Fisher’s exact test was used to test if a gene set demonstrated enrichment for genes involved in any gene ontology (GO) biology process. The significance level was set at a Bonferroni corrected *p*<0.05 (after correction for 825 independent GO biology process terms).

**References**

1. Huan T, Zhang B, Wang Z, Joehanes R, Zhu J, et al. (2013) A systems biology framework identifies molecular underpinnings of coronary heart disease. Arterioscler Thromb Vasc Biol 33: 1427-1434.

2. Joehanes R, Ying S, Huan T, Johnson AD, Raghavachari N, et al. (2013) Gene expression signatures of coronary heart disease. Arterioscler Thromb Vasc Biol 33: 1418-1426.

3. Boardman AE, Hui BS, Wold H (1981) The partial least squares-fix point method of estimating interdependent systems with latent variables. Communications in statistics-theory and methods 10: 613-639.

4. Abecasis GR, Cardon LR, Cookson WO, Sham PC, Cherny SS (2001) Association analysis in a variance components framework. Genet Epidemiol 21 Suppl 1: S341-346.

5. Hofman A, van Duijn CM, Franco OH, Ikram MA, Janssen HL, et al. (2011) The Rotterdam Study: 2012 objectives and design update. Eur J Epidemiol 26: 657-686.

6. Harries LW, Hernandez D, Henley W, Wood AR, Holly AC, et al. (2011) Human aging is characterized by focused changes in gene expression and deregulation of alternative splicing. Aging cell 10: 868-878.

7. Kirkendall WM, Feinleib M, Freis ED, Mark AL (1981) Recommendations for human blood pressure determination by sphygmomanometers. Subcommittee of the AHA Postgraduate Education Committee. Stroke 12: 555A-564A.

8. Hense H, Kuulasmaa K, Zaborskis A, Kupsc W, Tuomilehto J (1989) Quality assessment of blood pressure measurements in epidemiological surveys. The impact of last digit preference and the proportions of identical duplicate measurements. WHO Monica Project [corrected]. Revue d'épidémiologie et de santé publique 38: 463-468.

9. Price AL, Patterson NJ, Plenge RM, Weinblatt ME, Shadick NA, et al. (2006) Principal components analysis corrects for stratification in genome-wide association studies. Nat Genet 38: 904-909.

10. Stegle O, Parts L, Durbin R, Winn J (2010) A Bayesian framework to account for complex non-genetic factors in gene expression levels greatly increases power in eQTL studies. PLoS Comput Biol 6: e1000770.
